# Biodiversity drives the choice; linguistic diversity fine-tunes the direction: Ethnofloral megadiversity in the Mexican ethnobotany

**DOI:** 10.1371/journal.pone.0347334

**Published:** 2026-06-18

**Authors:** Andrea Martínez-Ballesté, Renata Sõukand, Carlos Martorell, Diana Flores- Camargo, Raivo Kalle, Julia Prakofjewa, Alejandro Casas

**Affiliations:** 1 Jardín Botánico, Instituto de Biología, Universidad Nacional Autónoma de México, Mexico City, Mexico; 2 Department of Environmental Sciences, Biocultural Diversity Lab, Informatics, and Statistics, Ca’ Foscari University of Venice, Venezia, Italy; 3 Savoirs, Environnement et Sociétés - SENS, Centre de Coopération Internationale en Recherche Agronomique pour le Développement, Montpellier, France; 4 Departamento de Ecología y Recursos Naturales, Facultad de Ciencias, Universidad Nacional Autónoma de México, Mexico City, Mexico; 5 Laboratory AMAP, Centre de Coopération Internationale en Recherche Agronomique pour le Développement, Montpellier, France; 6 Department of Folkloristics, Estonian Literary Museum, Tartu, Estonia; 7 Instituto de Investigaciones en Ecosistemas y Sustentabilidad, Universidad Nacional Autónoma de México, Morelia, Michoacán, México; University of Pernambuco: Universidade de Pernambuco, BRAZIL

## Abstract

People around the world have developed distinctive sets of useful plants, known as ethnofloras, which comprise a significant portion of Earth’s biodiversity. However, little is known about the factors that determine the composition of these collections and how different groups use plants. These differences may increase with geographic distance and linguistic separation due to barriers to communication and a lack of similarity in locally-available species. Using published ethnobotanical information, we analyzed the divergence in the composition of wild-species and the ways plants are used among 22 Mexican ethnic groups that use 2,855 species. We standardized plant use into ten categories and recorded them for each plant species and ethnic group. Each ethnic group uses a very large number of species (α-diversity), but few species are shared with other groups. Consequently, species turnover (β-diversity) between ethnic groups is very high. As expected, geographic distance fostered high differentiation in species composition of ethnofloras, probably reflecting differences in wild floras. Linguistic proximity promoted ethnoflora composition similarity, suggesting that communication plays a role in shaping the set of plants that are used. Geographic proximity also promotes similarity in how plants are used, though it is unclear whether language also plays a role. This suggests that language barriers are quite permeable. Social interactions and the use of Spanish as a *lingua franca* may favor the convergence of uses. This study provides a novel analysis of ethnofloras. It emphasizes the value of cultural and biological diversity and their importance in shaping ethnobotanical heritage.

## Introduction

The set of useful plants known and used by human groups, i.e., ethnofloras, can inform us about how cultural groups perceive and utilize nature’s diversity. The composition of ethnoflora differs between ethnic groups, reflecting the variety of ecosystems and the languages through which the knowledge that gives meaning and order to this diversity is transmitted [1]. The number of plant species that are collectively comprised in the world’s ethnofloras is enormous [2]. Globally, it is estimated that between 20% and 30% of the world’s described wild species (c.a. 280,000) are used for food [3]. Barron et al. [4] report 31,100 wild plants that are used worldwide, and Pironon et al. [2] compiled a global database of 35,687 wild, introduced or cultivated species. This diversity stems from two factors: many cultural groups use large numbers of species and different groups contribute different species to the global pool. These two contributions to the diversity of the world’s biocultural heritage correspond to what is known in ecology as alpha and beta diversity, respectively, [5] and are driven by the variety of available local species and exchange between human groups.

Intercultural variation between ethnofloras has been attributed to two related aspects. First, there are biological factors, such as plant traits, as well as the ecological contexts and floristic similarities of the inhabited geographic space. On the other hand, social drivers such as cultural affinities and mechanisms of knowledge transmission play a role [6,7]. Ecological approaches to ethnoflora composition focus on species accessibility and abundance (the ecological apparency hypothesis) [8,9]. Ethnofloristic compositional differences should thus reflect the decrease in similarity of wild floras with increasing geographic distance [10]. Regarding social drivers of differentiation, linguistic affinities imply relationships between ethnic groups and are used as indicators of intracultural relations. Language disseminates knowledge and can create barriers between linguistically unrelated groups [11]. Geographic distance may also hinder the communication of cultural traits [12]. Therefore, ethnic groups that speak related languages with high levels of mutual intelligibility or that occupy nearby territories, may have more similar ethnofloras due to knowledge diffusion. Furthermore, linguistically-related groups have inherited needs and knowledge from their common ancestors, resulting in greater similarity in their ethnofloras.

In addition to differences in ethnoflora composition, cultural groups also differ in how they use plants. A species may be used for one or more purposes [13,14]. The most commonly reported uses in ethnofloras are medicinal, followed by food and ornamental uses [15–17]. Intercultural differentiation in plant use is limited by plant traits that determine a plant’s utility. For example, wood thermal attributes determine plant’s use as fuel [18], and its bioactive properties determine its use as a medicinal plant [19]. Cultural membership may also drive differentiation by favoring certain uses over others (the protection hypothesis) [7,13,20,21]. Geographical and linguistic barriers, which affect ethnofloristic composition, are likely to drive differentiation in use. Thus, nearby ethnic groups or those that speak related languages are expected to use shared plant species similarity.

Thus, linguistic relationships can be considered a proxy for ethnofloras’ cultural affinities, while geographic separation reveals cultural and floristic variations [6]. However, geographic and linguistic factors are correlated. An ethnic group is one that shares a common cultural identity expressed through a common language [22], and related languages tend to be clustered in geographically close regions [6]. Therefore, similarities between nearby ethnic groups cannot be immediately attributed to geographic or linguistic proximity. Instead, the contribution of different drivers must be statistically disentangled [12].

Certain regions of the planet are hotspots of biocultural diversity, with high levels of both biological and linguistic diversity [23,24]. Wild-collected plants are the most abundant species in ethnofloras [16]. The richness of wild species used is particularly high in the tropical regions, as well as in some temperate regions (e.g., China, the Himalayas, Eastern Europe, and the eastern U.S.A) [2]. Twelve countries contain a large proportion of the world’s species richness and are home to 54% of the world’s living languages [25,26]. Mexico is one of these countries, being the convergence of two very rich biocultural regions, Mesoamerica, and Aridamerica. This region includes 11 linguistic families, which are organized into 68 main linguistic groups, making Mexico the fifth most linguistically-diverse country [25]. Mexico is also home to 23,314 species of vascular plants [27], representing one-third of the flora of the Americas [28]. Sixty-five percent of the country is occupied by mountain systems that facilitate a continuous turnover of species, increasing the country’s ecological *β* diversity [29]. Almost all types of vegetation found worldwide (deserts, tropical and temperate forests, grasslands, and high-altitude páramo) are found in Mexico [30]. Mexico has a long tradition of ethnobotanical studies and has one of the most complete sets of biocultural information in Latin America. The number of wild-collected species reported with some use represents 21% (approximately 4,900 species) of the national species pool [16] Thus, Mexico is one of the most dynamic places to study the use and management of flora, as well as how the species composition and uses of ethnofloras are structured in the context of high biological and linguistic diversity.

In this study we analyzed the ethnofloras’ divergences among 22 Mexican ethnic groups.

The objective was to evaluate 1) the similarity in the composition of wild plant species used by different ethnic groups, and 2) the similarity in the uses attributed to those species. These dimensions may be positively associated with either geographic or linguistic proximity. Geographic proximity reflects greater similarity in the available flora in spatially close regions, while linguistic proximity reflects shared cultural ancestry and/or greater ease of knowledge transmission among linguistically related groups. Although geographic and linguistic proximity are themselves spatially correlated, their effects can be considered partially independent and statistically disentangled.

## Methods

### Data collection

For four decades, information on useful plants species in Mexico has been continuously compiled and curated in a database using publicly available data from theses, books, and articles. This database, known as BADEPLAM (Base de Datos Etnobotánicos de Plantas Mexicanas), is one of the earliest and most comprehensive biocultural-information projects in Latin America. It is currently coordinated by us at the Botanical Garden of the Institute of Biology, at the National Autonomous University of Mexico (UNAM) [16].

Records of wild plant species and their uses, as assigned by ethnic groups, were obtained from information collected in BADEPLAM. Since plant use in Mexico has not been studied with the same intensity for all ethnic groups, those with few records in BADEPLAM were removed from the analysis to avoid misclassification in the cluster and dissimilarity analyses [31]. Only those with more than forty records were retained: Mayo (486 records), Tarahumara (592), Guarijío (267), Tepehuán (117), Pápago (41), Nahua (627), Maya (1572), Lacandón (135), Tzeltal (430), Tzotzil (611), Huasteco (382), Mixe (43), Zoque (106), Totonaco (582), Cuicateco (264), Mixteco (474), Ixcateco (200), Zapoteco (182), Chinanteco (205), Otomí (683), Purépecha (57), and Seri (410). For these groups, 8466 records, corresponding to 2855 wild plant species in 193 botanical families, were obtained. This information comes from 86 bibliographic research sources published mainly between 1980 and 2017 (75 sources) and 11 sources from the period 1935–1979 (S1 File). BADEPLAM updates the nomenclature and taxonomy of the species found in all sources, following the World Checklist of Vascular Plants (WCVP) [32], and the lists of vascular plants in Mexico [27,28].

### Data analysis

The average alpha diversity ($\overset{―}{\alpha }$) was estimated based on the number of species used by each ethnic group. Overall species turnover among ethnicities was obtained as (*β*) diversity as $\frac{\gamma }{\overset{―}{\alpha }}$ following Whittacker [5]. In this case, gamma (γ) was obtained from the total number of species used by the 22 ethnic groups.

We used the Jaccard index to measure pairwise dissimilarities of species composition in ethnofloras (S1 Script, [Data set. *SppEtnia_Jaccard.csv*]. Zenodo). To estimate differentiation in species use, we first standardized the reported uses into ten categories as proposed by Salick and coworkers [33]: a) food for animals, b) food for humans, c) environmental, d) fuel, e) construction, f) fiber, g) medicine, h) chemicals, i) cultural, and j) other. Species could have more than one use. For each species used by a pair of ethnic groups, calculated Jaccard dissimilarity using these data, and then calculated the average of this index for all shared species (S2 Script, [Data set. *usos_Jaccard.csv*]. Zenodo).

To visualize the dissimilarities, we used Sammon’s non-linear mapping (nMDS), which uses a two-dimensional configuration to reduce the stress value [34]. This was done for both compositional and use dissimilarities.

Using Google Earth Pro (Copyright 2025 Google LLC), we measured the geographic distance between points located approximately in the center of each ethnic group’s territory as reported by the “Atlas de los Pueblos Indígenas de México” (https://atlas.inpi.gob.mx/pueblos-indigenas/ accessed October 2024) ([Data set. *geographic distance.csv*]. Zenodo). The linguistic distance was based on the Ethnologue linguistic classification (https://www.ethnologue.com/ accessed October 2024). According to this source, each ethnic group speaks a language belonging to one of seven language families (Uto-Aztecan, Mayan, Mixe-Zoquean, Totonacan, Otomanguean, Tarascan, and an isolated language). These language families correspond to level 1 of the classification and are further divided into three hierarchical levels of mutual-intelligibility (Subgroups levels 2, 3 and 4) (Table 1). Distance values ranging from 1 to 5 were assigned based on the number of hierarchical levels separating one language from another ([Data set. *lingua distance.csv*]. Zenodo). Linguistic-distance values of 1 were assigned to languages belonging to the same level-4 group (e.g., Tarahumara and Guarijíos, Maya and Lacandón, Tzeltal and Tzotzil, Cuicateco and Mixteco). Languages in the same level-3 group were assigned a distance value of 2 (e.g., Otomi vs. Chinantec); a distance value of 3 was assigned to languages in the same level-2 group (e.g., Papago vs. Nahua), and a distance value of 4 was assigned to languages in the same family (level 1, e.g., Zapoteco vs. Chinanteco). A value of 5 is assigned to languages belonging to different families (e.g., Mayo vs. Maya, Maya vs. Mixe, and Zoque vs. Totonaco).

**Table 1:** Linguistic distance between ethnic groups. Hierarchical classification of the languages spoken by 22 ethnic groups in Mexico based on Ethnologue (https://www.ethnologue.com/). Level 1 shows the name of the language family; the following names correspond to subgroups of hierarchical levels 2 to 4. The last column lists the name of each ethnic group’s language. The colors indicate their relationship in the hierarchical classification from level 1 to level 4. Same colors indicate languages that share the same levels.

Family	Subgroups			Language
Level 1	Level 2	Level 3	Level 4	
Uto Aztecan	South	Taracahitic	Cahitan	Mayo
			Tarahumaran	Tarahumara
				Guarijío
		Pimic	Tepehuan	Tepehuán
			Tohono O’odham	Pápago
		Corachol-Aztecan	Core Nahua	Náhuatl
Mayan	Yucatecan-Core Mayan	Yucatecan	Yucatec-Lacandon	Maya
				Lacandón
		Core Mayan	Cholan-Tzeltalan	Tzeltal
				Tzotzil
	Huastecan	Huastec		Huasteco
Mixe-Zoquean	Mixean	Oaxaca Mixean		Mixe
	Zoquean	Chiapas Zoquean		Zoque
Totonacan				Totonaco
Otomanguean	Eastern	Amuzgo-Mixtecan	Mixtecan	Cuicateco
				Mixteco
		Popolocan-Zapotecan	Popolocan	Ixcateco
			Zapotecan	Zapoteco
	Western	Oto-Pame-Chinantecan	Chinantecan	Chinanteco
			Oto-Pamean	Otomí
Tarascan				Purépecha
Isolate				Seri

We used Mantel’s partial correlation test to assess the correlation between ethnofloristic composition or plant use and our two explanatory variables: the geographic distance and the linguistic differences between ethnic groups. Using partial correlations allowed us to separate the effects of geography and linguistics by removing their mutual correlation. These analyses were conducted using the vegan package [35] for R [36].

Since ethnic groups showed significant differences both in terms of the number of species recorded (range: 29–660) and in the number of studies where the information was obtained (range: 1–19), we performed three additional analyses to determine whether such differences in sampling effort, rather than true trends in the data, could explain our results. First, we repeated the above analyses using Simpson’s dissimilarity index instead of Jaccard’s. If two ethnic groups used the same plants, but differed in sampling intensity, the less studied group would have a subset of the species recorded for the more studied group. Under these conditions, Simpson’s index correctly indicates that both ethnicities use their plants identically, providing a dissimilarity measure insensitive to sampling intensity. Second, we subdivided the dataset into three sets of ethnic groups based on the number of species they used: 1–165 species (*n* = 8 ethnicities), 166–330 species (*n* = 10) and 331–660 species (*n* = 4). We then calculated the same Mantel partial correlations based on Jaccard’s index for each set as before. Since we performed three tests on the same hypothesis using different datasets, we can summarize the evidence from all three tests in a single test using Fisher’s method. This test integrates the evidence that each individual Mantel test provides against the null hypothesis (i.e., its *P*-value) into a *χ*^2^ test [37]. Note that each of the individual correlations on which the Fisher method is based is calculated from a dataset in which the disparity in species richness between ethnic groups has been minimized, reducing potential bias arising from different sampling efforts. Third, we used the same procedure but divided ethnic groups into three sets according to the number of studies in our sample: 1–2 studies (n = 8), 3–4 studies (n = 9) and more than 5 studies (n = 5).

## Results

On average, each ethnic group used an alpha diversity of $\overset{―}{\alpha }$ = 240 species (range: 29–660 species). There was high species turnover between cultural groups, with each ethnic group using only one-twelfth (*β* = 11.9) or 8% of the total recorded wild species diversity (*γ* = 2,855 species). Only 21 species were used by nine or more ethnic groups. In contrast, 1733 species were used by only one ethnic group, and 566 species were used by two groups (S1a-b Fig). Accordingly, the Jaccard’s dissimilarity values between ethnic groups in the reported wild species composition were high (range: 0.8 to 1, with one exception, S1 Table). This dissimilarity showed a positive and significant partial Mantel correlation with geographic distance (*r* = 0.3943, *p* = 0.0005 after controlling for language) and with linguistic distance between ethnicities, though the correlation was weaker (*r* = 0.2666, *p* = 0.0006 after controlling for geography). All three of the procedures used to test whether the previous results could be due to disparities in sampling-effort produced the same results (Table 2), indicating that the correlations are robust to differences in sampling.

**Table 2:** Relationship between geographic and linguistic distances and species composition and use categories based on three alternative methods to remove sampling-effort bias: using Simpson dissimilarity or grouping ethnicities to reduce their differences in the number of used species or number of studies consulted.

		All, Simpson dissimilarity		Grouped by number of species		Grouped by number of studies	
Data	Distance measure	*r*	*P*	*χ* ^2^	*P*	*χ* ^2^	*P*
Species	Geographic	0.39	< 0.001	25.2	< 0.001	16.3	0.012
	Linguistic	0.27	< 0.001	13.8	0.031	14.7	0.022
Use	Geographic	0.33	0.001	13.2	0.039	17.0	0.009
	Linguistic	0.10	0.051	4.43	0.618	10.0	0.122

**Fig 1:**
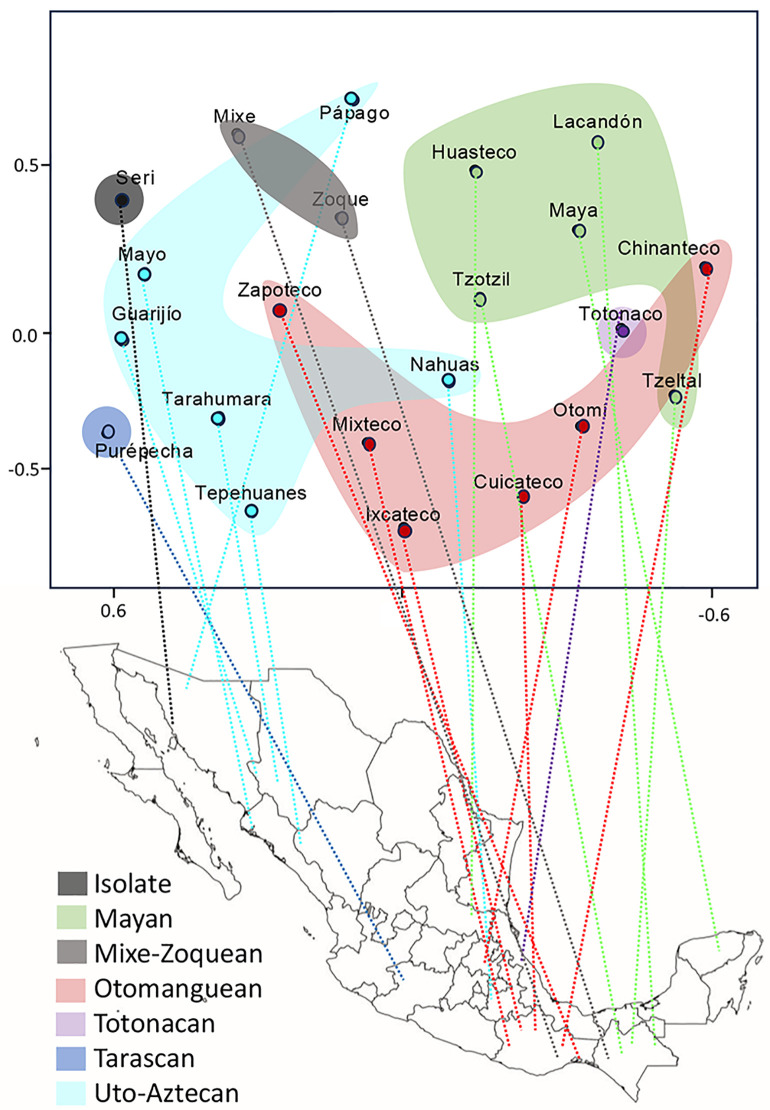
Ordination plot nMDS (non-linear Sammon mapping) of ethnic groups as a function of the species composition (stress = 0.119, Bray-Curtis). Colored polygons correspond to linguistic families. Lines indicate the approximate geographic center of each ethnic group’s range. The Mexico political map of administrative divisions states adapted from simplemaps.com [https://simplemaps.com/gis/country/mx#admin1] under a CC BY 4.0 license..

In the nMDS plot, ethnic groups cluster together when they are geographically close and share the same language family (Fig 1). For example, the Mayo and Guarijío ethnic groups, which inhabit near each other in the northwestern part of the country and belong to the same Uto-Aztecan language family had the least dissimilarity in their species composition (dissimilarity = 0.674). Ethnic groups that live close to each other but do not belong to the same linguistic family share more species than ethnic groups of the same linguistic family but live far apart. For example, the Seri (language isolate) and the Mayo (Uto-Aztecan) live close to each other and share more species (dissimilarity = 0.837), whereas the Mayo and the Nahua (both Uto-Aztecan) live far apart and share fewer species (dissimilarity = 0.920). However, there are exceptions to this general trend. For example, the Mixe and Zoque ethnic groups, which belong to the Mixe-Zoquean linguistic family, are geographically close to each other and do not share any species (dissimilarity = 1).

Most of the species had one (63%) or two use-categories (21%), while the remaining species had three to eight different uses (16%). The largest percentage of species are used for medicine, followed by those used plants for food, construction, and fodder. Other uses (environmental, cultural use, fuel, fiber, chemical, and others) account for 24% of the remaining records (S2 Fig). Although the dissimilarity in the use given to the wild species shared by each pair of ethnic groups was large, it was smaller than the dissimilarity in the species composition (range 0.5–1, S2 Table). Dissimilarity in plant use increased with the geographic distance (Mantel partial correlation *r* = 0.329, *p* = 0.001 after controlling for language), and, marginally, with linguistic distance (*r* = 0.101, *p* = 0.051 after controlling for geography). All procedures used to assess whether these results were due to differences in sampling-effort confirmed the correlation between use and geographic distance. However, the evidence for a correlation between use and linguistic distance was only marginally significant in the analysis based on Simpson’s index (Table 2)

**Fig 2:**
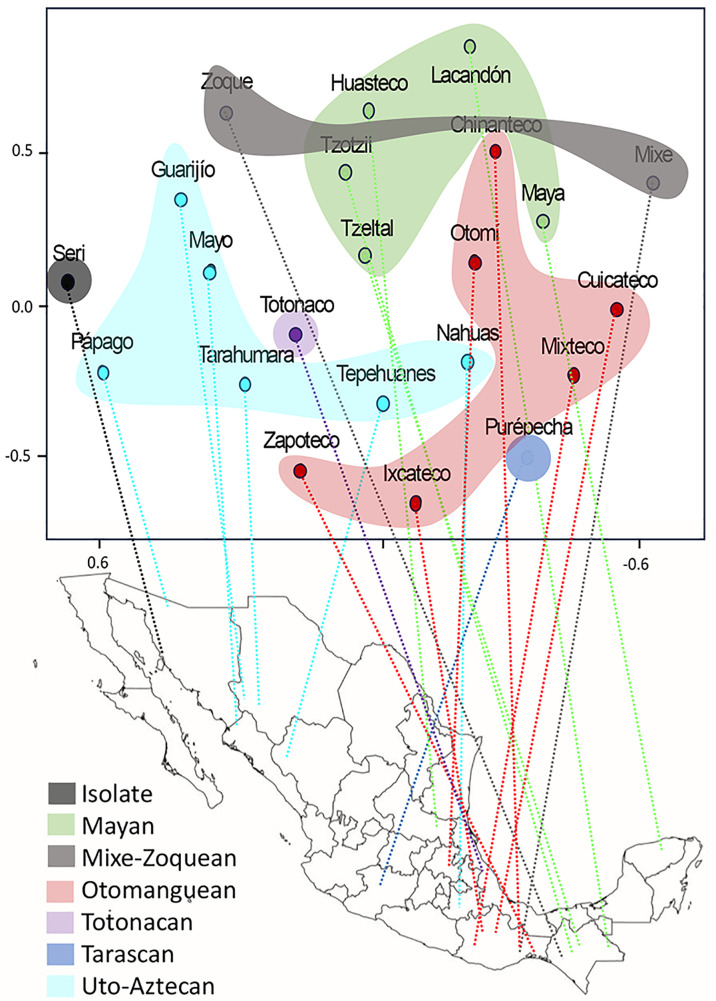
Ordination plot nMDS (Sammon’s non-linear mapping) of ethnic groups as a function of the uses (stress = 0.098, Bray-Curtis). Colored polygons correspond to linguistic families. Lines indicate the approximate geographic center of each ethnic group’s range. The Mexico political map of administrative divisions states adapted from simplemaps.com [https://simplemaps.com/gis/country/mx#admin1] under a CC BY 4.0 license.

Geographically and linguistically close ethnic groups use plants similarly. However, the groups they form (nMDS plot) are less compact than those observed in the species composition analysis (Fig 2). For example, the Maya are closer to ethnic groups that speak Otomanguean and Mixe-Zoquean languages than to other nearby Maya groups because their plant use is more similar.

## Discussion

Collectively, all the ethnic groups in this study used a high number of species, comprising 2855 species, and an average of 240 species per group. However, this is likely to be an underestimation of the Mexico’s significant biocultural legacy of traditional knowledge because the published literature consists of incomplete records. The main contributor to this substantial heritage is species turnover between ethnic groups. According to the Jaccard index, dissimilarity was high, and the *β* diversity of the ethnofloras revealed that each group used only 8% of the total number of species. The underlying geographic distribution of wild-plant biodiversity played a critical role in the composition of ethnofloras, as was reported for Nepal [6]. The two biogeographic kingdoms occurring in Mexico (Holarctic and Neotropical) cause differences in large portions of the country’s north and south. However, the topographic, geological, and climatic complexity multiplies the diversity of habitats in surprising ways [38] and causes a continuous exchange of wild species over short geographic distances (*β* diversity) [29]. Other studies in Mexico have shown high divergence in species composition among geographically close ethnic groups inhabiting extremely different natural environments due to sharp climatic and topographic gradients [39–43].

### Floristic composition of the ethnofloras

Understanding the determinants of *β* diversity is most important, given its significant contribution to Mexican biocultural heritage. As expected, ethnofloras were more similar among geographically close ethnic groups. Although less important, linguistic relatedness was also associated with the ethnoflora composition. Ethnic groups living in close proximity use more species in common because the flora available to them tends to be similar whether or not they belong to the same linguistic family [44]. Groups living far apart share few species because they inhabit biomes with completely different types of vegetation. For example, the Maya use plants from the tropical forests in southern Mexico, while the Papago use a different set of species in the deserts of northern Mexico.

Since related languages tend to occur in close proximity [11], the same factors that drive the relationship between ethnoflora similarity and geographic distance could arguably be invoked to explain similarities within linguistic families. However, a linguistic effect was still detected after the effect of geographic distance was removed, indicating that groups speaking related languages are associated in the analysis through the use the same species. This may reflect the permeability of linguistic barriers as mutual intelligibility increases. Languages belonging to the same family have common ancestors [45], so ethnic groups with greater linguistic relatedness may inherit a greater proportion of common plants from a shared past when language was not a barrier to communication. Thus, similarities within linguistic groups may reflect a faster transmission of knowledge [46] and the cultural conservatism of a shared heritage [45]. Studies of 12 ethnic groups in Nepal reveal similar patterns of medicinal flora due to linguistic kinship and geographical proximity, especially among groups inhabiting similar floristic environments [6].

### How uses are shared across cultural groups

The types of uses assigned by ethnic groups to the shared wild species are more similar when the groups are closer together than when they are farther apart. Since the dissimilarity in use was measured using species that are available to each pair of ethnic groups, the differences cannot be explained by the ethnofloras’ species turnover (*β* diversity) with distance. Therefore, it appears that it is communication between neighboring ethnic groups that drives use similarities. As expected, there was a positive correlation between linguistic distance and differentiation in uses. However, this effect was marginal, suggesting that the barrier between ethnic groups of different language families is permeable and increases consensus on how plants are used. Cultural groups are not closed social entities, and contact mechanisms exist between them [44]. Geographic proximity facilitates encounters between people from different communities through various social mechanisms. For example, local and regional markets provide spaces for sharing knowledge about plant diversity and its uses [47]. In Mexico, markets are long-standing institutions where people buy and sell regional products [48]. These spaces promote encounters between communities of different ethnic affiliations, and Spanish is often used in commercial transactions [49]. Even occasional events, such as intercultural marriages between people from neighboring villages can be enough to allow information about plant uses to cross language barriers, and then to spread rapidly. Currently, social media plays an important role in disseminating knowledge about plant uses of among cultural groups [7].

Some ethnic groups without linguistic association or geographic ties use certain species in similar ways. Ethnic groups with a cultural consensus on the use of certain species, find biological or ecological properties in them that satisfy certain needs [50], thus preserving knowledge of specific uses that has proven to solve cultural needs [51]. For example, the bioactive compounds present in certain species have facilitated consensus among cultural groups that assign medicinal uses to these plants [52,53]. In other cases, flavors generate cultural heritage for food plants used as spices in traditional cuisines [54,55], as is the case with the use of chili (*Capsicum annuum* L.) in the various expressions of Mexican cuisines.

We acknowledge that the temporal heterogeneity of the bibliographic sources compiled over several decades could introduce some bias into the interpretation of our results, since ethnofloristic knowledge and species use can change due to situations that alter ecological, socioeconomic, and cultural processes [56]. In this study, temporal variation was not assessed because we did not have repeated measurements from enough ethnic groups. However, considering that the majority (87%) of our citations are concentrated in a brief historical period of four decades (1980–2017), we might assume that there would not be as many changes.

## Conclusion

In this study, we found that the species that comprise the ethnofloras are more similar due to geographic and linguistic proximity. Geographic patterns of floristic diversity in Mexico contribute to the existence of very different ethnofloras with negligible species composition similarity. Each ethnic group shares few species with others, resulting in a very heterogeneous use of wild species diversity, even among ethnic groups that live close to each other and are linguistically related. Conversely, we found more consensus among ethnic groups regarding the uses of common species, perhaps because linguistic differences were not a significant barrier. In spaces of social interaction, such as markets or social networks, distances between ethnic groups are reduced due to a common *lingua franca*, Spanish, or convergence of uses occurs due to the characteristics of the species themselves. This study provides a broad ethnobotanical perspective on the relationship between biological and cultural diversity in Mexico by analyzing the structure of its ethnoflora. It emphasizes the value of cultural and biological diversity in shaping the ethnobotanical heritage.

## Supporting information

S1 File List of references. References that support the biocultural information collected in BADEPLAM for each ethnic group. References and uses are included. Use categories: food-animal (F_ANIM); food-human (F_HUMA); environmental (ENVIR); fuels (FUELS); construction (CONST); fibers (FIBER); medicines (MEDIC); chemicals (CHEMI); cultural uses (CUL_USE); and other (OTHER). Use categories: food-animal (F_ANIM); food-human (F_HUMA); environmental (ENVIR); fuels (FUELS); construction (CONST); fibers (FIBER); medicines (MEDIC); chemicals (CHEMI); cultural uses (CUL_USE); and other (OTHER).
(DOCX)

S1 Table Jaccard dissimilarity matrix. Dissimilarity values in composition species between ethnofloras.
(DOCX)

S2 Table Jaccard dissimilarity matrix. Dissimilarity values of the uses between ethnofloras.
(DOCX)

S1 Fig Wild species shared by the ethnic groups studied. (a) Species used by nine or more ethnic groups. Lantana camara, Guazuma ulmifolia, and Ricinus communis were the species shared by the most cultural groups. (b) The number of species shared between ethnic groups. Most of the species (1,733) are used by a single ethnic group, while only a few species are used by many different cultural groups.
(DOCX)

S2 Fig Percentage of species by use category. Species used as medicine outnumber other uses. The use categories are a) food-animal (F_ANIM), b) food-human (F_HUMA), c) environmental (ENVIR), d) fuels (FUELS), e) construction (CONST), f) fibers (FIBER), g) medicines (MEDIC), h) chemicals (CHEMI), i) cultural uses (CUL_USE), and j) other (OTHER).
(DOCX)

S1 Script Script to calculate Jaccard dissimilarity and cluster on species composition. (DOCX)

S2 Script Script to calculate Jaccard dissimilarity and cluster on the uses. (DOCX)
