# Supplementary material for: Biodiversity drives the choice; linguistic diversity fine-tunes the direction: Ethnofloral megadiversity in the Mexican ethnobotany
Source: PLoS One. 2026 Jun 18;21(6):e0347334. doi: 10.1371/journal.pone.0347334 (PMC13278395; doi:10.1371/journal.pone.0347334)
Supplement: S1 File — References that support the biocultural information collected in BADEPLAM for each ethnic group. References and uses are included. Use categories: food-animal (F_ANIM); food-human (F_HUMA); environmental (ENVIR); fuels (FUELS); construction (CONST); fibers (FIBER); medicines (MEDIC); chemicals (CHEMI); cultural uses (CUL_USE); and other (OTHER). Use categories: food-animal (F_ANIM); food-human (F_HUMA); environmental (ENVIR); fuels (FUELS); construction (CONST); fibers (FIBER); medicines (MEDIC); chemicals (CHEMI); cultural uses (CUL_USE); and other (OTHER). (DOCX) [file pone.0347334.s001.docx]

**S1. list of references**

*Cuicateco*.- Solis-Rojas L. (2006) Etnoecología Cuicateca en San Lorenzo Pápalo, Oaxaca. Tesis de Maestría. Universidad Nacional Autónoma de México (CONST; CUL_USE; CHEMI; ENVIR; F_ANIM; F_HUMA; FUELS; MEDIC).

*Chinanteco*.- Carrillo C. (2002) Las plantas en la vida de los pueblos de la Chinantla Baja. Tesis de Licenciatura. Universidad Nacional Autónoma de México (CONST; CUL_USE; ENVIR; F_HUMA; MEDIC; OTHER); Lipp F.J. (1971) Ethnobotany of the Chinantecas Indians, Oaxaca, México. Econ. Bot. 25(3):234-244 (CONST; CUL_USE; CHEMI; F_HUMA; MEDIC); Martin G.J. (1996) Comparative ethnobotany of the Chinantec and Mixe of the Sierra Norte, Oaxaca, Mexico. Tesis de Doctorado. Universidad de California (CONST; ENVIR; F_ANIM; F_HUMA; FUELS; MEDIC); Shapiro G. Colectas de campo (F_HUMA).

*Guarijío*.- García Ruiz I. & Linares Linares A. (2012) Árboles y arbustos de la cuenca del río Tepalcatepec. (Michoacán y Jalisco, México) para uso urbano. Instituto Politécnico Nacional, 304 p. (F_HUMA); Vázquez-García J.A., Cházaro-Basañez M.J., Nieves-Hernández G., Vargas-Rodríguez Y.L., Vázquez-García M. & Flores-Macías A. (2004) Serie Fronteras de Biodiversidad. Universidad de Guadalajara, 181 p. (CHEMI); Yetman D. (2002) The Guarijios of the Sierra Madre. Hidden people of Northwestern Mexico. UNM Press. 270 p. (CONST; CUL_USE; CHEMI; ENVIR; F_ANIM; F_HUMA; FIBER; FUELS; MEDIC; OTHER).

*Huasteco*.- Alcorn J. (1983) Dynamics of hustec ethnobotany resources. Perception and resources management at Teenek Tsabaal, México. Tesis de Doctorado. Austin University of Texas (CONST; CUL_USE; CHEMI; ENVIR; F_ANIM; F_HUMA; FIBER; FUELS; MEDIC; OTHER); Cilia López V.G., Aradillas C. & Díaz-Barriga F. (2015). Las plantas comestibles de una comunidad indígena de la Huasteca Potosina, San Luis Potosí. Entreciencias: Diálogos En La Sociedad Del Conocimiento, 3(7):143-152 (F_HUMA); García Ruiz I. & Linares Linares A. (2012) Árboles y arbustos de la cuenca del río Tepalcatepec. (Michoacán y Jalisco, México) para uso urbano. Instituto Politécnico Nacional, 304 p. (FUELS).

*Ixcateco*.- Rangel-Landa S. & R. Lemus (2002) Aspectos etnobotánicos y ecológicos de los recursos vegetales entre los Ixcatecos de Santa María Ixcatlán, Oaxaca, México. Tesis de Licenciatura. Universidad Michoacana de San Nicolás de Hidalgo (CONST; CUL_USE; CHEMI; ENVIR; F_ANIM; F_HUMA; FUELS; MEDIC).

*Lacandón*.- Duran-Fernández A. (1999) Estructura y etnobotánica de la selva alta perennifolia de Naha, Chiapas. Tesis de Maestría. Universidad Nacional Autónoma de México (CONST; CUL_USE; CHEMI; ENVIR; F_ANIM; F_HUMA; FIBER; FUELS; MEDIC; OTHER).

*Maya*.- Anderson E.N., Cauich-Canul J., Dzib A., Flores-Guido S., Islebe G., Medina-Tzuc F., Sánchez-Sánchez O. & Valdez-Chale P. (2005) Las plantas de los Mayas: etnobotánica en Quintana Roo, México. El Colegio de la Frontera Sur 206 p. (CONST; CUL_USE; CHEMI; ENVIR; F_ANIM; F_HUMA; FUELS; MEDIC); Barrera Marín A., Barrera-Vázquez A. & López-Franco R.M. (1976) Nomeclatura etnobotánica Maya: una interpretación taxonómica. Colección Científica 6. INAH-SEP. México. 537 p. (CONST; CUL_USE; CHEMI; ENVIR; F_ANIM; F_HUMA; FIBER; FUELS; MEDIC; OTHER); Caballero J. (1983) Colectas de campo (F_HUMA); Caballero J. (1992) Maya homegardens: Past, present and future. Etnoecológica. 1(1):35-54 (CONST; CHEMI; ENVIR; F_ANIM; F_HUMA; FIBER; MEDIC); Correa-Cano M.E. (2004) Los recursos vegetales en el paisaje fragmentado generado por la agricultura itinerante: un estudio de caso en el área Maya yucateca de México. Tesis de Licenciatura. Universidad Nacional Autónoma de México (CONST; CUL_USE; CHEMI; ENVIR; F_ANIM; F_HUMA; FUELS; MEDIC; OTHER); Estrada-Lugo E.I.J., Velazco-Te S., Bello-Baltazar E., Macario-Mendoza P.A., Segundo-Cabello A. & Sánchez-Pérez L. (2011) El rancho Maya. En: Bello-Baltazar E. y E.I.J. Estrada-Lugo (Comp.). Cultivar el territorio Maya: conocimiento y organización social en el uso de la selva. El Colegio de la Frontera Sur, pp. 99-131 (CONST; CUL_USE; ENVIR; F_ANIM; F_HUMA; MEDIC); Flores-Guido J.S., Tun-Garrido J., Ortiz-Díaz J.J. & Kantún-Balam J. (2010) Plantas usadas en cercas vivas en la península de Yucatán. Universidad Autónoma de Yucatán. 216 p. (ENVIR); Flores-Guido S. (1998) Etnobotánica de las Leguminosas en la Península de Yucatán. Uso y Manejo entre los Mayas. Tesis Doctoral. Universidad Nacional Autónoma de México (CONST; ENVIR; F_ANIM; F_HUMA; FUELS; MEDIC); Herrera-Castro N., Gómez-Pompa A., Cruz-Kuri L. & Flores J.S. (1993) Los huertos familiares mayas en X-uilub, Yucatán. Aspectos generales y estudio comparativo entre la flora de los huertos familiares y la selva. Biótica 1:19-36 (CONST; ENVIR; F_ANIM; FUELS; MEDIC); Hostettler U. (1996) Milpa agriculture and economic diversification. Socioeconomic change in a Maya peasant society of central Quintana Roo, 1900-1990s. Doctoral Dissertation, University of Berne, Switzerland (CONST; F_HUMA; MEDIC); La Torre-Cuadros M.A. & Islebe G.A (2003) Traditional ecological knowledge and use of vegetation in southeastern Mexico: a case study from Solferino, Quintana Roo Biodiversity and Conservation, 12(12): 2455-2476 (CONST; CUL_USE; ENVIR; F_HUMA; MEDIC; OTHER); Macario-Mendoza P.A. & Sánchez-Pérez L.C. (2011) Recolección de plantas: uso forestal tradicional y extracción forestal comercial en una comunidad maya del centro de Quintana Roo, pp. 213-236 (CONST); Martínez-Romero M.M., Castro Ramírez A.E. & Macario Mendoza P.A. (2011) Disponibilidad y aprovechamiento de bejucos artesanales en la zona Maya de la Biosfera de Sian Ka' an. En: Bello-Baltazar E. et al. (comp.). Cultivar el territorio Maya: conocimiento y organización social en el uso de la selva. El Colegio de la Frontera Sur, pp. 237-264 (CUL_USE); Martínez-Romero M.M., Castro-Ramírez A.E., Macario P. & Fernández J.C. (2004) Use and availability of craft vines in the influence zone of the Biosphere reserve Sian Ka'an, Quintana Roo, Mexico. Econ. Bot. 58(1):83-97 (CUL_USE); Sanabria O.L. (1986) El uso y manejo forestal en la comunidad de Xul, en el sur de Yucatán. Fasc. 2 191 p. (CONST; CUL_USE; CHEMI; ENVIR; F_ANIM; F_HUMA; FUELS; MEDIC; OTHER); Sánchez-González M.C. (1991) Uso y manejo de la leña en X-uilub, Yucatán. Tesis de Maestría. Universidad Nacional Autónoma de México (CHEMI; F_ANIM; F_HUMA; FUELS; MEDIC); Sierra-Huelsz J.A. (2017) Colectas de campo (CONST); Sousa-Novelo N. (1950) Plantas alimenticias y plantas de condimento que viven en Yucatán. Instituto Técnico Agrícola Henequenero, 265 p. (F_HUMA).

*Mayo*.- Lara-Ponce E. & Quintero-Romanillo A.L. (2016) Plantas medicinales del norte de Sinaloa. Universidad Autónoma Intercultural de Sinaloa, 41 p. (CUL_USE; ENVIR; F_HUMA; MEDIC); Yetman D. & Van Devender T.R. (2002) Mayo ethnobotany: land, history and traditional knowledge in Northwest Mexico. University of California Press. 359 p. (CONST; CUL_USE; CHEMI; ENVIR; F_ANIM; F_HUMA; FIBER; FUELS; MEDIC; OTHER).

*Mixe*.- Martin G.J. (1996) Comparative ethnobotany of the Chinantec and Mixe of the Sierra Norte, Oaxaca, Mexico. Tesis de Doctorado. Universidad de California (CONST; ENVIR; F_HUMA; FUELS; MEDIC; OTHER).

*Mixteco*.- Martin G.J. (1996) Casas A., Viveros J.L. & Caballero J. (1994) Etnobotánica Mixteca: sociedad, cultura y recursos naturales en la Montaña de Guerrero. Consejo Nacional para la Cultura y las Artes e Instituto Nacional Indigenista. México. 366 p. (CONST; CUL_USE; CHEMI; ENVIR; F_ANIM; F_HUMA; FUELS; MEDIC; OTHER); Viveros J.L. & Casas A. (1985) Etnobotánica Mixteca: Alimentación y subsistencia en la montaña de Guerrero. Tesis de Licenciatura. Universidad Nacional Autónoma de México (CHEMI; F_HUMA).

*Nahuas*.- Basurto F. (1982) Huertos familiares en dos comunidades nahuas de la sierra norte de Puebla: Yancuictlalpan y Cuauhtapanaloyan. Tesis de Licenciatura. Universidad Nacional Autónoma de México (CUL_USE; F_ANIM); Basurto-Peña F., Castro-Lara D. & Martínez-Alfaro M.A. (2003) Edible begonias from the north of Puebla, Mexico. Econ. Bot. 57(1):48-53 (F_HUMA); Caballero-Salas L. (1984) Plantas comestibles utilizadas en la sierra norte de Puebla por Totonacos y Nahuas. Tesis de Licenciatura. Universidad Nacional Autónoma de México (ENVIR; F_HUMA; MEDIC); Canales M., Hernández T., Caballero J., Romo de Vivar A., ÁvilaG., Durán A. & Lira R. (2005) Informant consensus factor and antibacterial activity of the medicinal plants used by the people of San Rafael Coxcatlán, Puebla, México. Journal of Ethnopharmacology, 97(3): 429-439 (MEDIC); Cedillo Portugal E. (1990) Las plantas útiles del Municipio de Tepoztlán, Morelos. Tesis de Maestría. Universidad Nacional Autónoma de México (CONST; CUL_USE; CHEMI; ENVIR; F_HUMA; FUELS; MEDIC); Cifuentes E. & Ortega M.A. (1990) Herbolaria y tradiciones etnomédicas en un pueblo Nahua. Universidad Nacional Autónoma de México. 146 p. (MEDIC); García Ruiz I. & Linares Linares A. (2012) Árboles y arbustos de la cuenca del río Tepalcatepec. (Michoacán y Jalisco, México) para uso urbano. Instituto Politécnico Nacional, 304 p. (ENVIR); Juárez-Vázquez, M. D, Carranza-Álvarez C., Alonso-Castro A.J., González-Alcaraz V.F., Bravo-Acevedo E., Chamarro-Tinajero F.J. & Solano E. (2013) Ethnobotany of medicinal plants used in Xalpatlahuac, Guerrero, México. Journal of Ethnopharmacology, 148: 521–527 (MEDIC); Martínez-Alfaro M.A., Evangelista V., Mendoza M., Morales G., Toledo G. & Wong A. (1995) Catálogo de plantas útiles de la Sierra Norte de Puebla, México. Cuadernos del Instituto de Biología 27:9-303 (F_HUMA); Mota-Cruz C. (2007) Plantas comestibles en la Sierra Negra de Puebla, México. Tesis de Maestría. Colegio de Postgraduados (F_HUMA); Rodríguez-López T. (2003) Manejo y conservación de las plantas medicinales comerciales, en el Municipio de Copalillo, Guerrero. Tesis de Maestría. Universidad Nacional Autónoma de México (MEDIC); Rojas-Gutiérrez J.F., Diego-Pérez N., Amith J.D. & Bye R. (2011) Plantas comestibles hortícolas: una necesidad en la dieta de tres comunidades nahuas de la Cuenca Media del río Balsas, Guerrero. Universidad Nacional Autónoma de México, pp. 23-45 (F_HUMA); Rosas-López R. (2003) Estudio etnobotánico de San Rafael-Coxcatlán. Tesis de Licenciatura. Universidad Nacional Autónoma de México (CONST; CUL_USE; CHEMI; ENVIR; F_ANIM; F_HUMA; FUELS; MEDIC; OTHER); Smith-Oka V. (2008) Plants used for reproductive health by Nahua women in northern Veracruz, Mexico. Econ. Bot. 62(4):604-614 (MEDIC); Vásquez-Rojas M.C. (1986) El uso de plantas silvestres y semicultivadas en la alimentación tradicional en dos comunidades campesinas del sur de Puebla. Tesis de Licenciatura. Universidad Nacional Autónoma de México (F_HUMA; MEDIC); Vázquez-Rojas C. (1983) Colectas de campo (CONST; CHEMI; F_ANIM; F_HUMA; FUELS; MEDIC); Villa-Kamel J.A. (1991) Las plantas utilizadas en forma tradicional en la alimentación en una comunidad nahua del este del estado de Hidalgo. Tesis de Licenciatura. Universidad Nacional Autónoma de México (F_HUMA); Villalobos-Contreras G. (1994) Plantas comestibles de la Sierra Norte de Puebla: Xochitlán de Vicente de Suárez y Zapotitlán de Méndez. Tesis de Licenciatura. Universidad Nacional Autónoma de México (CHEMI; F_HUMA; MEDIC; OTHER); Villaseñor-Martínez R. (1988) Etnobotánica de plantas comestibles de dos comunidades: San Pablito y Xolotla, en la sierra norte de Puebla. Tesis de Licenciatura. Universidad Nacional Autónoma de México (F_HUMA).

*Otomí*.- Castro A.E. (1988) Estudio comparativo del conocimiento sobre plantas medicinales utilizadas en dos grupos étnicos del Municipio de Pahuatlán, Puebla. Tesis de Licenciatura. Universidad Nacional Autónoma de México (CONST; CHEMI; ENVIR; F_HUMA; FUELS; MEDIC); Galinier J. (1979) n' yuhu: Les indiens Otomis. Etudes Mesoamericaines - Serie II. 615 p. (CONST; CUL_USE; CHEMI; ENVIR; F_ANIM; F_HUMA; FUELS; MEDIC; OTHER); López-Gutiérrez B. N., Pérez-Escandón B.E. & Villavicencio Nieto M.A. (2014) Aprovechamiento sostenible y conservación de plantas medicinales en Cantarranas, Huehuetla, Hidalgo, México, como un medio para mejorar la calidad de vida en la comunidad. Botanical Sciences, 92 (3): 389-404 (MEDIC); Martínez-Alfaro M.A., Evangelista V., Mendoza M., Morales G., Toledo G. & Wong A. (1995) Catálogo de plantas útiles de la Sierra Norte de Puebla, México. Cuadernos del Instituto de Biología 27:9-303 (MEDIC); Peters C., Rosenthal J. & Urbina T. (1987) Otomi bark paper in Mexico: Commercialization of a pre-hispanic technology. Econ. Bot. 41(3):423-432 (OTHER); Rangel S. (1987) Etnobotánica de los agaves del Valle del Mezquital. Tesis de Licenciatura. Universidad Nacional Autónoma de México (CHEMI; F_ANIM; F_HUMA; MEDIC); Romero-Lazcano E., Santiago-Altamirano A. & Basilio-García C. (1999) Plantas medicinales y de otros usos de San Antonio el Grande, Huehuetla, Hidalgo, en yuhu (Otomí de la Sierra). Universidad Autónoma del Estado de Hidalgo (CONST; CHEMI; ENVIR; F_HUMA; MEDIC; OTHER); Villaseñor-Martínez R. (1988) Etnobotánica de plantas comestibles de dos comunidades: San Pablito y Xolotla, en la sierra norte de Puebla. Tesis de Licenciatura. Universidad Nacional Autónoma de México (F_HUMA).

*Pápago*.- Nabhan G.P. & Rea A. (1987) Plant domestication and folk-biological change: The upper Piman. Devil's Claw example. American Anthropologist 89:57-73 (F_HUMA); Nabhan G.P., Rea A.M., Reichhardt K.L., Mellink E. & Hutchinson C.F. (1982) Papago influences on habitat and biotic diversity: quitovac oasis ethnoecology. Journal of Ethnobiology 2(2): 124-143 (CUL_USE; F_ANIM; F_HUMA; FUELS; MEDIC; OTHER).

*Purépecha*.- Caballero J. & Mapes C. (1982) Colectas de campo (CONST; CUL_USE; CHEMI; ENVIR; F_HUMA; FUELS; MEDIC; OTHER); García Ruiz I. & Linares Linares A. (2012) Árboles y arbustos de la cuenca del río Tepalcatepec. (Michoacán y Jalisco, México) para uso urbano. Instituto Politécnico Nacional, 304 p. (CONST; CUL_USE; CHEMI; ENVIR; F_HUMA; FUELS; MEDIC)

*Seri*.- Felger R. & Moser M. (1985) People of the desert and sea. Ethnobotany of the seri indians. University of Arizona Press. 435 p. (CONST; CUL_USE; CHEMI; ENVIR; F_ANIM; F_HUMA; FUELS; MEDIC; OTHER).

*Tarahumara*.- Bennet W. & Zingg M. (1935) Los Tarahumaras. Una tribu India del norte de México. Instituto Nacional de Antropología e Historia. 605 p. (F_HUMA; MEDIC); Bye R. (1985) Medicinal plants of the Tarahumara Indians of Chihuahua, Mexico. In: R.A. Tyson y D.V. Elrick (eds.). Two mummies from Chihuahua: A multidiciplinary study. San Diego Museum. 19: 77-104 (CHEMI; MEDIC); Bye R.A. (1976) Ethnoecology of the Tarahumara of Chihuahua, Mexico. Tesis de Doctorado. Universidad de Harvard (F_HUMA; MEDIC); Mares-Trias A. (1982) Comida de los Tarahumaras. Don Burgess Mc Guíre, Chihuahua, Chi. 501 p. (CONST; CHEMI; F_ANIM; F_HUMA; FUELS; MEDIC; OTHER); Pennington, C.W. (1963) The Tarahumara of Mexico: Their environment and material culture. University of Utah Press. 267 p. (CONST; CUL_USE; CHEMI; ENVIR; F_ANIM; F_HUMA; FUELS; MEDIC).

*Tepehuanes*.- González-Elizondo M., López-Enriquez I.L., González-Elizondo M.S. & Tena-Flores J.A. (2004) Plantas medicinales del estado de Durango y zonas aledañas. Instituto Politécnico Nacional. 144 p. (MEDIC); Pennington, C.W. (1969) The Tepehuan of Chihuahua. Their material culture. University of Utah Press. 413 p. (CONST; CUL_USE; CHEMI; F_ANIM; F_HUMA; FUELS; MEDIC); Vázquez-García J.A., Cházaro-Basañez M.J., Nieves-Hernández G., Vargas-Rodríguez Y.L., Vázquez-García M. & Flores-Macías A. (2004) Serie Fronteras de Biodiversidad. Universidad de Guadalajara, 181 p. (CHEMI; F_HUMA; FIBER).

*Totonaco*.- Aparicio-Alegría B.A. & García E. (1995) Percepción botánica: la visión del mundo natural por los Totonacos de Zozocolco de Hidalgo, Veracruz, México. Tesis de Licenciatura. Universidad Nacional Autónoma de México (CONST; CUL_USE; ENVIR; F_ANIM; F_HUMA; FUELS; MEDIC); Caballero-Salas L. (1984) Plantas comestibles utilizadas en la sierra norte de Puebla por Totonacos y Nahuas. Tesis de Licenciatura. Universidad Nacional Autónoma de México (F_HUMA; MEDIC); Castro-Lara D. (2000) Etnobotánica y papel económico de cuatro especies de quelites en Tuxtla, Zapotitlán de Méndez. Puebla, Mex. Tesis de Licenciatura. Universidad Nacional Autónoma de México (F_HUMA); Cuevas J.A. (1991) Definición, aprovechamiento y conservación de recursos fitogéneticos en una comunidad indígena totonaca. Tesis de Maestría. Colegio de Postgraduados (CONST; CUL_USE; ENVIR; F_HUMA; FIBER); Espadas M. & G. Zita (1982) Contribución al conocimiento de la flora medicinal de los totonacos de la Sierra de Puebla (Tuzamapan de Galeana). Tesis de Licenciatura. Universidad Nacional Autónoma de México (F_HUMA; MEDIC); Kelly I. & A. Palerm (1952) The Tajin Totonac. Smithsonian Institution. Institute of Social Anthropology (CONST; CUL_USE; CHEMI; F_ANIM; F_HUMA; MEDIC); Martínez-Alfaro M.A. (1984) Medicinal plants used in a Totonac community of the Sierra Norte de Puebla: Tuzamapan de Galeana Puebla, Mexico. Journal of Ethnopharmacology, 11(2):203-221 (CONST; CUL_USE; ENVIR; F_ANIM; F_HUMA; FUELS; MEDIC); Martínez-Alfaro M.A., Evangelista V., Mendoza M., Morales G., Toledo G. & Wong A. (1995) Catálogo de plantas útiles de la Sierra Norte de Puebla, México. Cuadernos del Instituto de Biología 27:9-303 (F_ANIM; F_HUMA; MEDIC); Martínez-Murillo M. E. (1992) Especies vegetales como recurso energético de uso doméstico. Zozocolco de Hidalgo, Veracruz. Tesis de Licenciatura. Universidad Nacional Autónoma de México (F_HUMA; FUELS; MEDIC); Morales G. & Toledo G. (9187) Contribución a la flora medicinal tradicional del municipio de Coxquihui, Veracruz. Tesis de Licenciatura. Universidad Nacional Autónoma de México (CONST; CUL_USE; ENVIR; F_ANIM; F_HUMA; FUELS; MEDIC); Villalobos-Contreras G. (1994) Plantas comestibles de la Sierra Norte de Puebla: Xochitlán de Vicente de Suárez y Zapotitlán de Méndez. Tesis de Licenciatura. Universidad Nacional Autónoma de México (CHEMI; F_HUMA; MEDIC).

*Tzeltal*.- Berlin B., Breedlove D. & Raven P. (1974) Principles of the Tzeltal plant classification. An introduction to the botanical ethnography of a mayan speaking people of highland Chiapas. Academic Press. 660 p. (CONST; CUL_USE; CHEMI; ENVIR; F_HUMA; FIBER; FUELS; MEDIC; OTHER); Calderón-Cisneros A. (2001) Uso y acceso a los recursos forestales en una comunidad indígena: la leña en Amatenago del Valle, Chiapas, México. Tesis de Maestría. El Colegio de la Frontera Sur (FUELS); Gallardo M.C., Tellez H.M.C., Vargas V.M.S. & Vázquez G.L. (1983) Aspectos Etnobotánicos y Bacteriológicos en la Medicina Tradicional en los Altos de Chiapas. Tesis de Licenciatura. Universidad Nacional Autónoma de México (MEDIC).

*Tzotzil*.- Breedlove D.E. & Laughlin R.M (1993) The flowering of Man: A Tzotzil botany of Zinacantán. Smithsonian Contributions to Anthropology (CONST; CUL_USE; CHEMI; ENVIR; F_ANIM; F_HUMA; FUELS; MEDIC; OTHER); Gallardo M.C., Tellez H.M.C., Vargas V.M.S. & Vázquez G.L. (1983) Aspectos Etnobotánicos y Bacteriológicos en la Medicina Tradicional en los Altos de Chiapas. Tesis de Licenciatura. Universidad Nacional Autónoma de México (MEDIC).

*Zapoteco*.- Frei B. (1997) Medicinal ethnobotany of the Isthmus. Sierra Zapotecs (Oaxaca, Mexico) and biological-phytochemical investigation of selected medicinal plants. Marmota, Switzerland. 380 p. (MEDIC); Messer E. (1978) Zapotec plant knowledge: Classification, uses and comunication about plants in Mitla, Oaxaca. Memorias of the Museum of Anthropology University of Michigan. 10(5), 140 p. parte 2 (F_ANIM; F_HUMA; MEDIC); Saynes A. (2006) Colectas de campo (CONST; CHEMI; ENVIR; F_ANIM; F_HUMA; FIBER; FUELS; MEDIC; OTHER).

*Zoque*.- Isidro-Vázquez M.A. (1997) Etnobotánica de los Zoques de Tuxtla Gutiérrez, Chiapas. Instituto de Historia Natural, 125 p. (CONST; CUL_USE; CHEMI; ENVIR; F_ANIM; F_HUMA; FIBER; FUELS; MEDIC).
