# Supplementary figures and images for: Biodiversity drives the choice; linguistic diversity fine-tunes the direction: Ethnofloral megadiversity in the Mexican ethnobotany
Source: PLoS One. 2026 Jun 18;21(6):e0347334. doi: 10.1371/journal.pone.0347334 (PMC13278395; doi:10.1371/journal.pone.0347334)

S1 Fig.


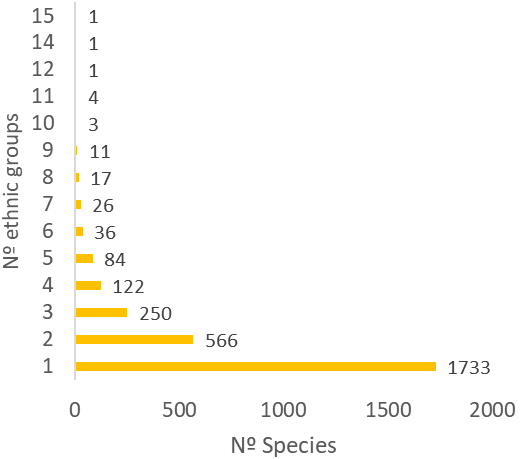

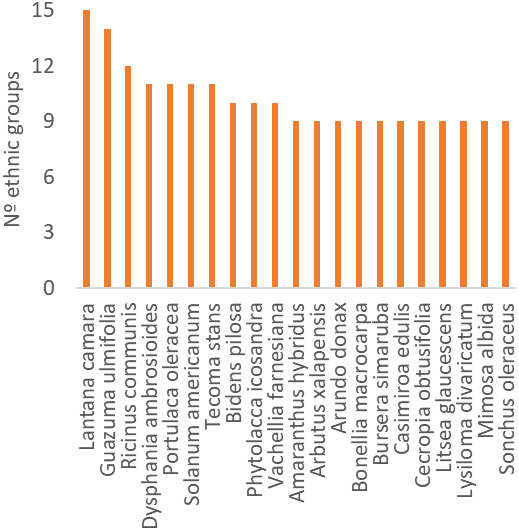


a

b

Supplement: S1 Fig — (a) Species used by nine or more ethnic groups. Lantana camara, Guazuma ulmifolia, and Ricinus communis were the species shared by the most cultural groups. (b) The number of species shared between ethnic groups. Most of the species (1,733) are used by a single ethnic group, while only a few species are used by many different cultural groups. (DOCX) [file pone.0347334.s004.docx]

S2 Fig.

**
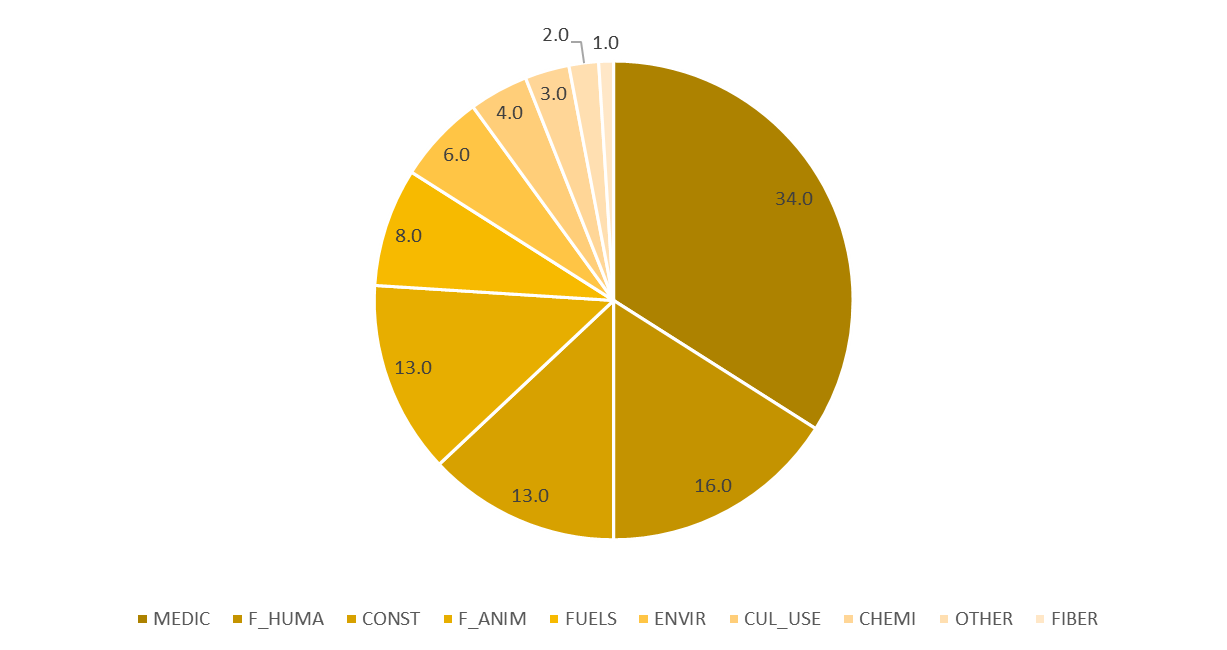
**

Supplement: S2 Fig — Species used as medicine outnumber other uses. The use categories are a) food-animal (F_ANIM), b) food-human (F_HUMA), c) environmental (ENVIR), d) fuels (FUELS), e) construction (CONST), f) fibers (FIBER), g) medicines (MEDIC), h) chemicals (CHEMI), i) cultural uses (CUL_USE), and j) other (OTHER). (DOCX) [file pone.0347334.s005.docx]
