# Supplementary material for: Biodiversity drives the choice; linguistic diversity fine-tunes the direction: Ethnofloral megadiversity in the Mexican ethnobotany
Source: PLoS One. 2026 Jun 18;21(6):e0347334. doi: 10.1371/journal.pone.0347334 (PMC13278395; doi:10.1371/journal.pone.0347334)
Supplement: S1 Script — (DOCX) [file pone.0347334.s006.docx]

**S1 Script**.

library(vegan)

dat=read.csv("SppEtnia_Jaccard.csv")

jacc=vegdist(t(dat[,2:23]),method="jaccard",binary=T)

plot(hclust(jacc,method="ward.D2"))
