# Supplementary material for: Biodiversity drives the choice; linguistic diversity fine-tunes the direction: Ethnofloral megadiversity in the Mexican ethnobotany
Source: PLoS One. 2026 Jun 18;21(6):e0347334. doi: 10.1371/journal.pone.0347334 (PMC13278395; doi:10.1371/journal.pone.0347334)
Supplement: S2 Script — (DOCX) [file pone.0347334.s007.docx]

**S2 Script**.

datos=read.csv("usos_ Jaccard.csv")

names(datos)[1]="sp"

datos[,1]=as.factor(datos[,1])

#this function calculates for a pair of ethnicities e1 and e2 the fraction of shared uses

#(out of all the uses reported) for shared each species, and returns the average for all

#the shared species.

sim2=function(e1,e2,dat=datos){

numusos=table(dat$sp)

nombres=levels(datos$sp)

numsp=length(nombres)

salida=1:numsp

for(nsp in 1:numsp){

subdat=datos[which(datos$sp==nombres[nsp]),c(e1+2,e2+2)]

esta=min(colSums(subdat))

salida[nsp]=ifelse(esta==0,NA,sum(subdat[,1]*subdat[,2])/numusos[nsp])

}

names(salida)=nombres

salida

}

#This function calculates the use dissimilarity matrix for all pairs of ethnicities

#using the function sim2

matsim2=function(dat=datos){

net=dim(dat)[2]-2

nomet=names(dat[3:(net+2)])

salida=matrix(ncol=net,nrow=net)

colnames(salida)=nomet

rownames(salida)=nomet

for(i in 1:net){

for(j in 1:net){

salida[i,j]=1-mean(sim2(i,j),na.rm=T)

}

}

salida[which(salida=="NaN")]=1

as.dist(salida)

}

#obtain the matrix and conduct cluster analysis

dd2=matsim2()

plot(hclust(dd2,method="average"))
