# Supplementary material for: Biodiversity drives the choice; linguistic diversity fine-tunes the direction: Ethnofloral megadiversity in the Mexican ethnobotany
Source: PLoS One. 2026 Jun 18;21(6):e0347334. doi: 10.1371/journal.pone.0347334 (PMC13278395; doi:10.1371/journal.pone.0347334)
Supplement: Attachment — Submitted filename: RESPON~1.DOC [file pone.0347334.s009.DOC]

RESPONSE TO REVIEWERS AND EDITOR

**Editor comments:**

*Some relevant studies addressing closely related ideas are not cited in the introduction. It would be helpful to briefly discuss their main findings and clarify how your study advances beyond them. For example: https://doi.org/10.1098/rspb. 2013.2768; https://doi.org/10.1016/j.jep.2014.12.007

RESPONSE: We appreciate this suggestion; these articles were very valuable in improving our interpretation of the data. The articles are now cited in the introduction and in the discussion. Our works are relatively similar, so it helps us confirm the patterns we observed.

*In the paragraph presenting your hypotheses, the distinction between plant composition and similarity in use remains somewhat unclear. Currently, only the hypothesis concerning geographic proximity is explicitly formulated, whereas the expected effect of linguistic relatedness on species composition is not clearly stated. Please revise this section to make both aspects explicit and conceptually distinct.

RESPONSE: We made some phrasing changes to better formulate the hypotheses of geographical and linguistic proximity and the relationship with the two approaches of this study; species composition and species uses among ethnofloras. The changes made to the introduction, reinforcing the theoretical framework, also contribute to a better explanation of the hypotheses.

**Reviewer A**

*My recommendation is that the authors review and strengthen the theoretical framework in the introduction and add a more robust discussion of their results. I reiterate that the study is highly relevant and should be published, but I believe that the data are currently underutilized in the way the manuscript is presented.

RESPONSE: Thank you for your congratulations, and we hope we have improved the article with your valuable feedback.

Introduction

*The authors mention only briefly, at the end, that geographic proximity may serve in the study as a proxy for an environmental factor influencing plant selection, while linguistic proximity may serve as a proxy for a cultural factor. This was the way I was able to interpret the issue, although it is not clearly stated in the text. I strongly suggest that the authors expand the introduction to outline the mechanisms through which geographic proximity could actually reflect environmental similarity or, in some cases, facilitate human mobility and thus the sharing of information (we might be observing either a convergence or divergence evolutionary process, depending on the case).

The same applies to the linguistic perspective. What evolutionary aspects underlie this cultural proxy? Is it a shared past and the broader phenomenon whereby linguistic divergence occurred alongside the differentiation of other cultural traits, such as local ecological knowledge? Or is it rather the ease of communication in more recent times? In that case, what is the role of the currently adopted colonial language? In summary, I felt the lack of a more comprehensive introduction that would help the reader fully understand the phenomenon under study.

I strongly recommend that the authors consult literature along the following lines, which may help them to develop a deeper and more coherent argumentative framework:

RESPONSE: We appreciate your suggestions. The literature you and the editor shared helped us strengthen our theoretical framework on geographic and linguistic processes. Taking your observation into account, the introduction now includes information that better supports the theoretical framework explaining how geographic proximity and linguistic phylogeny, proxies for the cultural and floristic affinities are explained variations in the uses and species composition of ethnofloras.

Methods:

*What is the rationale for choosing a cutoff of 40 records for inclusion in the database? Is there a statistical justification for this choice?

RESPONSE: When a system (in this case an ethnic group) has very few data, a few unusual species can lead to severe misclassification (in cluster analyses) or to poor estimates of dissimilarity. Thus, it is common practice to eliminate such data (Jongman, R.H.G., Ter Braak, C.J. and Van Tongeren, O.F. eds., 1995. Data analysis in community and landscape ecology. Cambridge university press). This information and reference has been added to the main text.

*Another question: Did the authors consider reanalyzing the data by grouping together only those ethnic groups with a similar number of recorded species? Although the authors argue that the agreement between Simpson and Jaccard indices would demonstrate the absence of sampling bias, what methodological biases might still exist when comparing the dissimilarity of a group with 29 species to another with 660? Even if the 29 species are included within the set of 660, would they truly be considered completely similar? I recommend excluding outliers in terms of the number of species and re-running the analyses to verify whether dissimilarity decreases and how this might affect the results and discussion.

RESPONSE: This is a very nice idea and addresses also the concerns of reviewer C about incomplete sampling. We repeated our analyses after removing all ethnic groups with only one and with more than 5 studies and found the same results as with the Simpson index.

However, we can do better that this and take advantage of all our data without dropping information. Ethnicities can be grouped based on the number of species recorded for each one or the number of publications that we consulted. The Mantel correlations can then be calculated for each data subset. Because the subsets would thus have far less variability in terms of sampling effort, any bias that it may introduce in the correlations would be strongly reduced. Because the individual Mantel statistics are testing the same null hypothesis using different datasets, they can then be merged into a single test via the Fisher’s method (Gotelli, N.J. and Ellison, A.M. 2013. A Primer of Ecological Statistics. Sinauer, Sunderland; Shipley, B., 2016. Cause and correlation in biology: A user's guide to path analysis, structural equations and causal inference with R. Cambridge university press.). Again, such grouping by number of species or of publications produced the same results as the Mantel analysis based on the Simpson index. The fact that three different statistical procedures give the same outcome suggests that our results are in fact robust to incomplete-sampling bias. The methodological description and the results table were added to the main text.

* In addition, the authors should provide a clearer explanation of how the analysis of species composition and plant use categories was performed. Were these separated into information units such as plant–function pairs? The authors could include examples to help readers better understand how this categorization was carried out.

RESPONSE: We have provided a better description of the method and indicated the path in the repository (Zenodo) where the matrices can be consulted. Due to a misunderstanding regarding the PLOS ONE instructions, we did not provide open access to Zenodo. We will now provide a Google Drive link so that reviewers can anonymously examine the matrices, and if the article is accepted, access to Zenodo will be made public. We believe that once you have access to the matrices (SppEtnia_Jaccard.csv and usos_Jaccard.csv), it will be clear how the data were organized for the species composition and land use analyses.

Results:

*I found Table S1 difficult to interpret. Could the authors clarify what the names indicated at each level (e.g., “South,” “Pimic,” “Cahitan”) represent? Do they correspond to sub-branches or internal divisions within each language family?

RESPONSE: The language spoken by each ethnic group is classified into subgroups (levels 2 to 4) that belong to a main language family (level 1). This measures the degree of intelligibility of the languages. The names of each variant come from how these groups named their languages or how they are named according to the classification established by linguists. To be clearer, in the methods section we have added an explanation of this hierarchical division and also made improvements to the table and its caption. This table now includes a color-coded guide to represent the hierarchical relationship of the languages. We also considered placing the table within the main text so that the description of the linguistic distance estimation would be closer to the table used for the calculation.

Discussion:

*There are divergent ideas in the discussion that make it difficult to fully understand the phenomenon. On the one hand, the authors suggest a possible environmental selection effect (as I proposed earlier as a theoretical pathway for the introduction), arguing that geographically close localities tend to have more similar ethnofloras. However, the text later reaffirms that all ethnofloras are very different from each other, even those that are geographically close. It is also mentioned that nearby localities can be ecologically very distinct, which contradicts the initial explanation. Without considering these specificities, it becomes unclear what the real pattern revealed by the analyses is. Again, I suggest reconsidering the analyses by removing the outliers to verify whether this could help clarify the observed trends.

I also suggest that the discussion be further developed regarding the possibility that plant selection is driven more by the environment than by language (with language serving as a cultural proxy). Although this is not entirely clear, it seems to be the pattern supported by the study.

RESPONSE: We understand the confusion and have strived to present the arguments in the discussion more clearly. We believe that the improvements since the introduction help to better understand the effect of beta diversity, which shows the high percentage of turnover among ethnofloras because of Mexico's floristic characteristics. Therefore, the Jaccard dissimilarity values are high (0.8 to 1), indicating that the ethnofloras are not very similar. Nevertheless, the pattern is significant (as verified by Mantel's correlation using the four suggested methods), and we can say that the species composition of the ethnofloras is correlated with geographic and linguistic proximity, and the uses of species are correlated with geographic proximity and marginally with language. Therefore, in the discussion, we consider it important to explain the effects of the environment and language because, although to different degrees, both explain variations in species composition and uses.

To improve clarity, we made changes to the organization of the writing that we hope will help to better discuss the results.

*I also believe that the study lacks a more detailed discussion of the fact that differences in dissimilarity with respect to geographic and linguistic separation are more pronounced when analyzed at the level of the complete information unit (plant–use) compared to analyses based solely on species diversity.

RESPONSE: The reason for the greater similarity in the uses of species is that language is not a barrier and allows for greater consensus among ethnic groups regarding the uses they assign to species. This argument is found in the discussion between lines 359 and 363.

**Reviewer B**

Lines 63 to 66: When discussing biocultural knowledge and its relationship to alpha and beta diversity, there is no reference to support this. There are publications related to the topic.

RESPONSE: We didn't include a reference because we couldn't find one that presented a similar idea. In this case, we are proposing this analogy between alpha-beta ecological diversity and the way knowledge about biocultural diversity is distributed. However, in that section we have added a reference that explains the ecological terms alpha and beta.

Lines 118-125: It's important to mention the methodology used to obtain the information found in BADEPLAM. A reference alone isn't enough.

RESPONSE: Thank you for your observation. To address your comment, we have included information in the methodology that more clearly describes the process by which BADEPLAM's information is obtained. We have clarified that it has been a historical and ongoing process of compiling and curating data on useful plants, obtained from publicly available bibliographic sources. We also clarify that this database is currently under our management, and therefore we are responsible for its curation. The methodology section describes how the scientific names of the species are curated to keep the names up to date.

Lines 125-126: The methodology used to obtain the articles that were analyzed in the study is not mentioned. If the list of included studies was the result of a systematic review, it is important to include all stages of the process.

RESPONSE: We appreciate your feedback, as it helps us better communicate our methodology. The data used for this article was already captured and curated in BADEPLAM and comes from the articles listed in the supplementary section. The selection of bibliographic sources in BADEPLAM is the result of a historical and continuous search process that has been ongoing for 40 years. As mentioned in the previous response, this explanation is found in the method section, where we aim to explain how the people in charge of the database have selected their sources over this period.

**Reviewer C**

The manuscript is interesting and well written. However, several theoretical and methodological aspects are missing in order for it to be clear and to become an appropriate contribution for PLOS ONE. The authors are requested to address the following points:

1.The study analyzes changes in ethnofloras among human groups within Mexico. However, it does not compare its results with any other research conducted in other countries. It is necessary to present evidence of patterns observed regarding the turnover in the use of plant species at comparable scales, such as countries or regions. Accordingly, a substantial expansion of both the Introduction and Discussion sections is expected.

RESPONSE: We appreciate your observation and have placed greater emphasis on finding other research that has studied the intercultural variation of ethnofloras at the scale we did, incorporating cultural and geo-floristic processes. Research by Saslis-Lagoudakis, which we had not previously reviewed, particularly the work published in 2014, shares many attributes with our research. This article, along with others shared by the editor and reviewers, are cited in the introduction and discussion to enhance the theoretical framework and allow for comparisons with other regions such as Nepal or the Basque Country in Spain.

The analysis of floristic turnover in ethnofloras is a proposal we put forward in this article, but we have not found a similar analysis in another research. Saslis-Lagoudakis et al. (2014) estimate differences in the ethnic groups´ medicinal floras and floristic environments in Nepal using phylogenetic information of the flora, but not beta diversity. Therefore, we have no citations from other regions worldwide.

2. I consider the supplementary material insufficient. An essential element in any research article is the provision of sufficient methodological information to allow reproducibility. The “Supplementary list of references” (Supporting information S1.PDF) shows that a significant portion of the original sources are unpublished works corresponding to theses at different academic levels. Do the authors have the legal rights to use these sources, given that it is understood that they reproduce the extensive species lists compiled by those students? It is necessary that this ethical aspect be explicitly addressed by the authors and that it complies with PLOS ONE’s policies.

RESPONSE: We appreciate your observation, therefore the description of the information contained in the database (BADEPLAM) (see Methodology) explicitly states that the data obtained from theses, books and articles are public data. This is stated in the article:

“*For four decades, information of Mexican useful plants species has been continuously compiled and curated in a database using publicly available data in theses, books, and articles*.”

Regarding your concern about free access to data from published works, the copyright of literary works (including theses) protects their creative expression and original form in which the data is presented. However, copyright does not protect, among other things, the data itself or lists of data. Therefore, it is legitimate to collect species names and other data for a different purpose (https://www.copyright.gov/circs/circ01.pdf, https://www.copyright.gov/circs/circ33.pdf

3. The authors do not provide sufficient data in their supplementary material, or at least they do not do in a format that can be easily reviewed anonymously. The authors provide an annex (https://doi.org/10.5281/zenodo.17238925), although it is not possible to access it because doing so would compromise the anonymity of the reviewer. This may also be the case for future readers in the event the manuscript is published. It is therefore essential that the information be made openly available within the journal’s own platform. Furthermore, sufficient information must be provided so that the analyses can be replicated. In its current form, this point is unclear.

RESPONSE: We apologize for this inconvenience. We followed PLOS One's instructions by selecting the option that the data in the Zenodo repository would be available until publication. For this submission, we will temporarily make the data available to reviewers by sending a Drive link so they can access and view the data anonymously.

The Zenodo repository contains the absence-presence matrices necessary for the two dissimilarity analyses ([Data set. SppEtnia_Jaccard.csv and usos_Jaccard.csv]. Zenodo). It also includes the geographical and linguistic distance matrices used for the Mantel analyses ([Data set. geographic distance.csv and lingua distance.csv]. Zenodo). With this information, and by following the methodology outlined in the article and the R scripts provided in the supplementary material, it is possible to replicate the analyses.

4. It is essential to explicitly state the confidence intervals obtained for the ethnoflora analyses for each Indigenous group reported. It is not clear whether further sampling is needed or whether the data compiled from different sources provide adequate sampling effort. The sampling effort must be specified for each Indigenous group included in the analysis. Curves indicating sampling effort and confidence intervals for the results for each Indigenous group are required. This must also be discussed.

RESPONSE: Because we deal with ethnofloristic alpha-diversity, the use of accumulation curves is quite sensible but poses some serious problems. In any ethnographic study the number of useful species is almost certainly underestimated: some remain unrecorded because they are rarely used or mentioned. To solve this problem, different techniques have been developed based on how species accumulate per unit of sampling effort. This is extremely difficult to conduct in our case because we lack, for each study, the sampling effort (number of closed questionnaires, area sampled, or hours of interview or participant observation) is rarely published and is not likely to be comparable across studies. Moreover, a lot of ethnographic data is compiled in a way that effort is not measurable.

This leaves us with the number of publications consulted per ethnic group as the sole possible measure of sampling effort. However, this has the problem that the underlying true sampling effort for different studies study may be very different. Take for instance Tzotzils, for whom 586 species are reported in just one exhaustive two-volume publication, while for Tarahumaras we know of 299 species after consulting five different, less-detailed sources. Publications cannot be equated with sampling-effort units without much error.

Nevertheless, given the importance of the true number of species used per ethnic group, we considered it worthy to try such estimation based on the number of publications. Because of the small number of studies per ethnic group, fitting a parametric species-accumulation curve seems inappropriate. Instead, we used the non-parametric is the Chao1 index. We calculated also the standard deviation in the number of species reported per study as a measure of intra-ethnicity variation in sampling effort, and the ratio of Chao1 to the number of species reported as a measure of diversity subestimation (the larger the ratio, the more useful species not reported, and thus the larger subestimation in the number of species used). These are the results:

| Ethnic group | Number of publications | Number of species reported | Sampling-effort variation | Chao1 | subestimation |
| --- | --- | --- | --- | --- | --- |
| Mayo | 2 | 326 | 215.7 | 4836.2 | 14.8 |
| Mixteco | 2 | 182 | 101.1 | 549.8 | 3.0 |
| Pápago | 2 | 39 | 24.0 | 761.0 | 19.5 |
| Purépecha | 2 | 41 | 4.2 | 281.7 | 6.9 |
| Tzotzil | 2 | 586 | 412.2 | 57234.2 | 97.7 |
| Guarijío | 3 | 265 | 152.4 | 17557.3 | 66.3 |
| Huasteco | 3 | 250 | 140.6 | 31250.5 | 125.0 |
| Tepehuanes | 3 | 93 | 28.7 | 867.4 | 9.3 |
| Tzeltal | 3 | 297 | 156.0 | 6262.8 | 21.1 |
| Zapoteco | 3 | 125 | 40.5 | 7813.0 | 62.5 |
| Chinanteco | 4 | 149 | 41.3 | 2777.1 | 18.6 |
| Tarahumara | 5 | 299 | 71.4 | 563.2 | 1.9 |
| Otomí | 8 | 357 | 71.0 | 1461.9 | 4.1 |
| Totonaco | 11 | 310 | 31.3 | 689.5 | 2.2 |
| Maya | 19 | 660 | 101.3 | 1266.4 | 1.9 |
| Nahuas | 19 | 430 | 38.3 | 1253.0 | 2.9 |

We then conducted a multiple regression analysis to see if the subestimation decreases with the number of publications, as expected if the fraction of unrecorded species decreases as more studies are conducted, and how subestimation is affected by intra-ethnicity variation in sampling effort across publications. We found that such variation increases the subestimation (*F* = 4.99, *P* = 0.044), which is independent of the number of studies (*F* = 2.93, *P* = 0.111).

What this means is that the estimate of the number of species used is heavily inflated by the variation in sampling effort across studies. Consider Tzoztils: Chao1 indicates that there are nearly 100 unknown useful species per each species reported, indicating a huge subestimation of alpha diversity. Moreover, the estimated size of their ethnoflora is twice the number of species existing in all of Mexico. This absurd figure results solely from differences in sampling effort. One study reports the totality of the species we know that are used by Tzoztils, while the other reports a much smaller number and no new species. This results in a large number of singletons (species reported in only one study) which is the basic input of Chao1. This bias is likely to arise independent of whether Chao1 or any other procedure is used.

Given the impossibility to obtain sensible estimates for the total number of useful species based on the number of publications as sampling-effort measure, we have decided not to include the above results in the manuscript. However, the problem of alpha-diversity subestimation is now acknowledged in the main text (Discussion section). Given that the alpha diversity is already large using current data, further sampling would not change our conclusion that ethnic groups in Mexico use a great variety of plants.

Regarding the possible effect of incomplete sampling on beta diversity, we had already provided an analysis to address the possible biases due to differences in sampling effort (re-analysis of the data using Simpson dissimilarity). Following a suggestion of reviewer one and addressing your concerns, we have conducted further analyses controlling for the differences in number of published studies and in species numbers. Our conclusions are the same in the three different analyses. This means that biases related to sampling effort do not explain the patterns that we had reported in the manuscript with the possible exception of the use-linguistics correlation.
