# Supplementary material for: Biodiversity drives the choice; linguistic diversity fine-tunes the direction: Ethnofloral megadiversity in the Mexican ethnobotany
Source: PLoS One. 2026 Jun 18;21(6):e0347334. doi: 10.1371/journal.pone.0347334 (PMC13278395; doi:10.1371/journal.pone.0347334)
Supplement: Attachment — Submitted filename: Response to Reviewers2.docx [file pone.0347334.s010.docx]

RESPONSE TO EDITOR COMMENTS

First, some of the interpretative language in the discussion occasionally appears stronger than what the statistical analyses strictly allow. Given that the Mantel and partial Mantel tests assess correlations rather than causal relationships, I recommend carefully revising statements that imply determination or causality. Framing the results in terms of statistically supported associations would ensure greater conceptual precision and avoid overinterpretation.

**Response: Thank you for your observation. We have carefully reviewed the language of our interpretations in the discussion and softened it into two sentences (lines 339-340; 348-349) where we now explain the results as associations rather than causal relationships.**

Second, although the authors have made a commendable effort to address sampling effort concerns, particularly regarding alpha diversity, there remain important limitations inherent to the use of heterogeneous bibliographic sources collected over many decades. In this regard, I encourage the authors to explicitly consider the temporal dimension as a potential source of bias. Some areas may be represented primarily by older studies, while others are documented mostly by recent research. Likewise, for the same area, combining studies conducted in very different time periods may introduce a temporal bias, since ethnofloristic knowledge and species use can change substantially over time due to ecological transformations, socioeconomic shifts, or cultural dynamics. A more explicit acknowledgment of this temporal heterogeneity, and its possible implications for the observed patterns, would strengthen the discussion of limitations.

**Response:** **We understand your argument and agree that the differences in the years covered by the literature used can bias the interpretation. Since we do not have studies that are consistently repeated across different years for all ethnicities, it was acknowledged at the end of the discussion (lines 389-396) that we lack this type of data to analyze this temporal variation in this research. However, we also argued in the discussion that we could assume there are not many changes, as we found that 87% of the citations we used correspond to a modern four-decade period (1980-2017), and only 11 references cover the period from 1935 to 1979. This distribution of citations across the years was also explained in the methodology section (lines 166-167).**

Third, I would like to draw attention to the formulation of the hypothesis. In its current wording, the hypothesis appears to conflate distinct mechanisms within a single explanatory framework. However, these components operate through partially different processes and would benefit from clearer analytical separation. A more precise formulation would distinguish between (1) similarity in the composition of wild plant species used by different ethnic groups and (2) similarity in the uses attributed to those species. These dimensions may be positively associated with (i) geographic proximity, reflecting greater similarity in the available flora in spatially close regions, and (ii) linguistic proximity, reflecting shared cultural ancestry and/or greater ease of knowledge transmission among linguistically related groups. Importantly, as acknowledged in earlier sections of the Introduction, geographic and linguistic proximity are themselves spatially correlated. For this reason, their effects should be treated as partially independent and statistically disentangled. Clarifying the hypothesis along these lines would strengthen its conceptual coherence by explicitly separating ecological constraints from cultural transmission processes, distinguishing species composition from patterns of use, and aligning the theoretical expectations more closely with the analytical framework employed in the study.

**Response: We appreciate your suggestions for better explaining the objectives and hypotheses of this research. We have taken your suggestions into account, and the changes can be found at the end of the introduction (lines 137-145).**
