# Supplementary material for: Biodiversity drives the choice; linguistic diversity fine-tunes the direction: Ethnofloral megadiversity in the Mexican ethnobotany
Source: PLoS One. 2026 Jun 18;21(6):e0347334. doi: 10.1371/journal.pone.0347334 (PMC13278395; doi:10.1371/journal.pone.0347334)
Supplement: Attachment — Submitted filename: Reviewe.docx [file pone.0347334.s008.docx]

Lines 63-66: When discussing biocultural knowledge, which is related to alpha and beta diversity, no reference is mentioned to support this. Studies have been conducted that have analyzed this relationship.

Lines 118-125: It is important to mention the source of the information contained in BADEPLAM. The methodology must be included; a reference is not sufficient.

Lines 125-126: Mention the methodology used to obtain the articles analyzed in the study. If it was through a systematic review, all steps of the methodology must be included.
