# Supplementary material for: Biodiversity drives the choice; linguistic diversity fine-tunes the direction: Ethnofloral megadiversity in the Mexican ethnobotany
Source: PLoS One. 2026 Jun 18;21(6):e0347334. doi: 10.1371/journal.pone.0347334 (PMC13278395; doi:10.1371/journal.pone.0347334)
Supplement: Peer review history

# Peer review: Decision Letter 0

**DOI:** 10.1371/journal.pone.0347334.r001

**Authors:** Washington Ferreira Júnior

3 Nov 2025

PONE-D-25-53514

Biodiversity drives the choice; linguistic diversity fine-tunes the direction: Ethnofloral megadiversity in the Mexican ethnobotany.

PLOS ONE

Dear Dr. Martínez-Ballesté,

Thank you for submitting your manuscript to PLOS ONE. After careful consideration, we feel that it has merit but does not fully meet PLOS ONE’s publication criteria as it currently stands. Therefore, we invite you to submit a revised version of the manuscript that addresses the points raised during the review process.

Please submit your revised manuscript by Dec 18 2025 11:59PM. If you will need more time than this to complete your revisions, please reply to this message or contact the journal office at plosone@plos.org. When you're ready to submit your revision, log on to https://www.editorialmanager.com/pone/ and select the 'Submissions Needing Revision' folder to locate your manuscript file.

Please include the following items when submitting your revised manuscript:

- A rebuttal letter that responds to each point raised by the academic editor and reviewer(s). You should upload this letter as a separate file labeled 'Response to Reviewers'.
- A marked-up copy of your manuscript that highlights changes made to the original version. You should upload this as a separate file labeled 'Revised Manuscript with Track Changes'.
- An unmarked version of your revised paper without tracked changes. You should upload this as a separate file labeled 'Manuscript'.

If you would like to make changes to your financial disclosure, please include your updated statement in your cover letter. Guidelines for resubmitting your figure files are available below the reviewer comments at the end of this letter.

If applicable, we recommend that you deposit your laboratory protocols in protocols.io to enhance the reproducibility of your results. Protocols.io assigns your protocol its own identifier (DOI) so that it can be cited independently in the future. For instructions see: https://journals.plos.org/plosone/s/submission-guidelines#loc-laboratory-protocols. Additionally, PLOS ONE offers an option for publishing peer-reviewed Lab Protocol articles, which describe protocols hosted on protocols.io. Read more information on sharing protocols at https://plos.org/protocols?utm_medium=editorial-email&utm_source=authorletters&utm_campaign=protocols.

We look forward to receiving your revised manuscript.

Kind regards,

Washington Soares Ferreira Júnior

Academic Editor

PLOS ONE

Journal Requirements:

When submitting your revision, we need you to address these additional requirements.

1. Please ensure that your manuscript meets PLOS ONE's style requirements, including those for file naming. The PLOS ONE style templates can be found at https://journals.plos.org/plosone/s/file?id=wjVg/PLOSOne_formatting_sample_main_body.pdf and https://journals.plos.org/plosone/s/file?id=ba62/PLOSOne_formatting_sample_title_authors_affiliations.pdf

2. When completing the data availability statement of the submission form, you indicated that you will make your data available on acceptance. We strongly recommend all authors decide on a data sharing plan before acceptance, as the process can be lengthy and hold up publication timelines. Please note that, though access restrictions are acceptable now, your entire data will need to be made freely accessible if your manuscript is accepted for publication. This policy applies to all data except where public deposition would breach compliance with the protocol approved by your research ethics board. If you are unable to adhere to our open data policy, please kindly revise your statement to explain your reasoning and we will seek the editor's input on an exemption. Please be assured that, once you have provided your new statement, the assessment of your exemption will not hold up the peer review process.

3. Thank you for stating the following in the Acknowledgments Section of your manuscript:

AMB thanks Javier Caballero Nieto for opening the way to the compilation of ethnobotanical information from Mexico. CM thanks the financial support for a sabbatical leave from Dirección General de Asuntos del Personal Académico DGAPA-PASPA. This paper is the result of AMB's sabbatical leave, supported by the Universidad Nacional Autónoma de México, for the study of biocultural diversity.

We note that you have provided funding information that is not currently declared in your Funding Statement. However, funding information should not appear in the Acknowledgments section or other areas of your manuscript. We will only publish funding information present in the Funding Statement section of the online submission form.

Please remove any funding-related text from the manuscript and let us know how you would like to update your Funding Statement. Currently, your Funding Statement reads as follows:

CM recived financial support for a sabbatical leave from Dirección General de Asuntos del Personal Académico DGAPA-PASPA, UNAM

Please include your amended statements within your cover letter; we will change the online submission form on your behalf.

4. We note that Figure(s) 1 and 2 in your submission contain [map/satellite] images which may be copyrighted. All PLOS content is published under the Creative Commons Attribution License (CC BY 4.0), which means that the manuscript, images, and Supporting Information files will be freely available online, and any third party is permitted to access, download, copy, distribute, and use these materials in any way, even commercially, with proper attribution. For these reasons, we cannot publish previously copyrighted maps or satellite images created using proprietary data, such as Google software (Google Maps, Street View, and Earth). For more information, see our copyright guidelines: http://journals.plos.org/plosone/s/licenses-and-copyright.

We require you to either (1) present written permission from the copyright holder to publish these figures specifically under the CC BY 4.0 license, or (2) remove the figures from your submission:

a. You may seek permission from the original copyright holder of Figure(s) 1 and 2 to publish the content specifically under the CC BY 4.0 license.

We recommend that you contact the original copyright holder with the Content Permission Form (http://journals.plos.org/plosone/s/file?id=7c09/content-permission-form.pdf) and the following text:

“I request permission for the open-access journal PLOS ONE to publish XXX under the Creative Commons Attribution License (CCAL) CC BY 4.0 (http://creativecommons.org/licenses/by/4.0/). Please be aware that this license allows unrestricted use and distribution, even commercially, by third parties. Please reply and provide explicit written permission to publish XXX under a CC BY license and complete the attached form.”

Please upload the completed Content Permission Form or other proof of granted permissions as an "Other" file with your submission.

In the figure caption of the copyrighted figure, please include the following text: “Reprinted from [ref] under a CC BY license, with permission from [name of publisher], original copyright [original copyright year].”

b. If you are unable to obtain permission from the original copyright holder to publish these figures under the CC BY 4.0 license or if the copyright holder’s requirements are incompatible with the CC BY 4.0 license, please either i) remove the figure or ii) supply a replacement figure that complies with the CC BY 4.0 license. Please check copyright information on all replacement figures and update the figure caption with source information. If applicable, please specify in the figure caption text when a figure is similar but not identical to the original image and is therefore for illustrative purposes only.

The following resources for replacing copyrighted map figures may be helpful:

USGS National Map Viewer (public domain): http://viewer.nationalmap.gov/viewer/

The Gateway to Astronaut Photography of Earth (public domain): http://eol.jsc.nasa.gov/sseop/clickmap/

Maps at the CIA (public domain): https://www.cia.gov/library/publications/the-world-factbook/index.html and https://www.cia.gov/library/publications/cia-maps-publications/index.html

NASA Earth Observatory (public domain): http://earthobservatory.nasa.gov/

Landsat: http://landsat.visibleearth.nasa.gov/

USGS EROS (Earth Resources Observatory and Science (EROS) Center) (public domain): http://eros.usgs.gov/#

Natural Earth (public domain): http://www.naturalearthdata.com/

5. If the reviewer comments include a recommendation to cite specific previously published works, please review and evaluate these publications to determine whether they are relevant and should be cited. There is no requirement to cite these works unless the editor has indicated otherwise.

Additional Editor Comments :

Thank you for submitting your manuscript entitled “Biodiversity drives the choice; linguistic diversity fine-tunes the direction: Ethnofloral megadiversity in the Mexican ethnobotany” to *PLOS ONE*. The manuscript addresses an important question concerning the factors that shape ethnofloral diversity in Mexico. By analyzing published ethnobotanical data from 22 Indigenous groups, the study explores how divergences in wild plant species composition and uses increase with both geographic distance and linguistic separation. The reviewers found the topic relevant and the dataset rich, with strong potential to contribute to the field. However, all three reviewers agreed that revisions are required before the manuscript can be considered for publication.

Below you will find the detailed comments from Reviewers. In addition, I include a few suggestions that I believe could help strengthen the paper, particularly in the framing of the hypotheses and contextualization within the broader literature.

*Some relevant studies addressing closely related ideas are not cited in the introduction. It would be helpful to briefly discuss their main findings and clarify how your study advances beyond them. For example: https://doi.org/10.1098/rspb.2013.2768; https://doi.org/10.1016/j.jep.2014.12.007

*In the paragraph presenting your hypotheses, the distinction between plant composition and similarity in use remains somewhat unclear. Currently, only the hypothesis concerning geographic proximity is explicitly formulated, whereas the expected effect of linguistic relatedness on species composition is not clearly stated. Please revise this section to make both aspects explicit and conceptually distinct.

*Reviewer A

I congratulate the authors on their work. The study investigates how geographic proximity and linguistic relationships influence the composition and use of wild plants among 22 Indigenous groups in Mexico. The authors hypothesize that ethnofloras become more distinct with increasing geographic distance, and that culturally or linguistically related groups share more similar plant uses due to shared ancestral knowledge and interaction. Although the topic is highly interesting and the study presents extremely rich data and is well written, it lacks a stronger theoretical framework to demonstrate the expected relationship between geographic proximity, linguistic phylogeny, and ethnoflora.

My recommendation is that the authors review and strengthen the theoretical framework in the introduction and add a more robust discussion of their results. I reiterate that the study is highly relevant and should be published, but I believe that the data are currently underutilized in the way the manuscript is presented.

Below, I provide my specific comments on each topic.

Introduction

The authors mention only briefly, at the end, that geographic proximity may serve in the study as a proxy for an environmental factor influencing plant selection, while linguistic proximity may serve as a proxy for a cultural factor. This was the way I was able to interpret the issue, although it is not clearly stated in the text. I strongly suggest that the authors expand the introduction to outline the mechanisms through which geographic proximity could actually reflect environmental similarity or, in some cases, facilitate human mobility and thus the sharing of information (we might be observing either a convergence or divergence evolutionary process, depending on the case).

The same applies to the linguistic perspective. What evolutionary aspects underlie this cultural proxy? Is it a shared past and the broader phenomenon whereby linguistic divergence occurred alongside the differentiation of other cultural traits, such as local ecological knowledge? Or is it rather the ease of communication in more recent times? In that case, what is the role of the currently adopted colonial language? In summary, I felt the lack of a more comprehensive introduction that would help the reader fully understand the phenomenon under study.

I strongly recommend that the authors consult literature along the following lines, which may help them to develop a deeper and more coherent argumentative framework:

Currie, Thomas E.; Mace, Ruth. The evolution of ethnolinguistic diversity. Advances in Complex Systems, [S. l.], v. 15, n. 01n02, p. 1150006, 2012. DOI: 10.1142/S0219525911003372.

Hua, Xia; Greenhill, Simon J.; Cardillo, Marcel; Schneemann, Hilde; Bromham, Lindell. The ecological drivers of variation in global language diversity. Nature Communications, [S. l.], v. 10, n. 1, p. 2047, 2019. DOI: 10.1038/s41467-019-09842-2.

Moore, Joslin L.; Manne, Lisa; Brooks, Thomas; Burgess, Neil D.; Davies, Robert; Rahbek, Carsten; Williams, Paul; Balmford, Andrew. The distribution of cultural and biological diversity in Africa. Proceedings of the Royal Society of London. Series B: Biological Sciences, [S. l.], v. 269, n. 1501, p. 1645–1653, 2002. DOI: 10.1098/rspb.2002.2075.

Methods:

What is the rationale for choosing a cutoff of 40 records for inclusion in the database? Is there a statistical justification for this choice?

Another question: Did the authors consider reanalyzing the data by grouping together only those ethnic groups with a similar number of recorded species? Although the authors argue that the agreement between Simpson and Jaccard indices would demonstrate the absence of sampling bias, what methodological biases might still exist when comparing the dissimilarity of a group with 29 species to another with 660? Even if the 29 species are included within the set of 660, would they truly be considered completely similar? I recommend excluding outliers in terms of the number of species and re-running the analyses to verify whether dissimilarity decreases and how this might affect the results and discussion.

In addition, the authors should provide a clearer explanation of how the analysis of species composition and plant use categories was performed. Were these separated into information units such as plant–function pairs? The authors could include examples to help readers better understand how this categorization was carried out.

Results:

I found Table S1 difficult to interpret. Could the authors clarify what the names indicated at each level (e.g., “South,” “Pimic,” “Cahitan”) represent? Do they correspond to sub-branches or internal divisions within each language family?

Discussion:

There are divergent ideas in the discussion that make it difficult to fully understand the phenomenon. On the one hand, the authors suggest a possible environmental selection effect (as I proposed earlier as a theoretical pathway for the introduction), arguing that geographically close localities tend to have more similar ethnofloras. However, the text later reaffirms that all ethnofloras are very different from each other, even those that are geographically close. It is also mentioned that nearby localities can be ecologically very distinct, which contradicts the initial explanation. Without considering these specificities, it becomes unclear what the real pattern revealed by the analyses is. Again, I suggest reconsidering the analyses by removing the outliers to verify whether this could help clarify the observed trends.

I also suggest that the discussion be further developed regarding the possibility that plant selection is driven more by the environment than by language (with language serving as a cultural proxy). Although this is not entirely clear, it seems to be the pattern supported by the study.

I also believe that the study lacks a more detailed discussion of the fact that differences in dissimilarity with respect to geographic and linguistic separation are more pronounced when analyzed at the level of the complete information unit (plant–use) compared to analyses based solely on species diversity.

My final suggestion is that the authors consider resubmitting the work in the future, incorporating a stronger theoretical framework and a more thorough discussion to fully leverage the richness of the data and results.

*Reviewer B

Lines 63 to 66: When discussing biocultural knowledge and its relationship to alpha and beta diversity, there is no reference to support this. There are publications related to the topic.

Lines 118-125: It's important to mention the methodology used to obtain the information found in BADEPLAM. A reference alone isn't enough.

Lines 125-126: The methodology used to obtain the articles that were analyzed in the study is not mentioned. If the list of included studies was the result of a systematic review, it is important to include all stages of the process.

*Reviewer C

The manuscript is interesting and well written. However, several theoretical and methodological aspects are missing in order for it to be clear and to become an appropriate contribution for PLOS ONE. The authors are requested to address the following points:

1.The study analyzes changes in ethnofloras among human groups within Mexico. However, it does not compare its results with any other research conducted in other countries. It is necessary to present evidence of patterns observed regarding the turnover in the use of plant species at comparable scales, such as countries or regions. Accordingly, a substantial expansion of both the Introduction and Discussion sections is expected.

2. I consider the supplementary material insufficient. An essential element in any research article is the provision of sufficient methodological information to allow reproducibility. The “Supplementary list of references” (Supporting information S1.PDF) shows that a significant portion of the original sources are unpublished works corresponding to theses at different academic levels. Do the authors have the legal rights to use these sources, given that it is understood that they reproduce the extensive species lists compiled by those students? It is necessary that this ethical aspect be explicitly addressed by the authors and that it complies with PLOS ONE’s policies.

3. The authors do not provide sufficient data in their supplementary material, or at least they do not do in a format that can be easily reviewed anonymously. The authors provide an annex (https://doi.org/10.5281/zenodo.17238925), although it is not possible to access it because doing so would compromise the anonymity of the reviewer. This may also be the case for future readers in the event the manuscript is published. It is therefore essential that the information be made openly available within the journal’s own platform. Furthermore, sufficient information must be provided so that the analyses can be replicated. In its current form, this point is unclear.

4. It is essential to explicitly state the confidence intervals obtained for the ethnoflora analyses for each Indigenous group reported. It is not clear whether further sampling is needed or whether the data compiled from different sources provide adequate sampling effort. The sampling effort must be specified for each Indigenous group included in the analysis. Curves indicating sampling effort and confidence intervals for the results for each Indigenous group are required. This must also be discussed.

This is an interesting study; however, it is considered that several aspects, such as those mentioned above, must be further developed in greater depth.

[Note: HTML markup is below. Please do not edit.]

Reviewers' comments:

Reviewer's Responses to Questions

**Comments to the Author**

1. Is the manuscript technically sound, and do the data support the conclusions?

The manuscript must describe a technically sound piece of scientific research with data that supports the conclusions. Experiments must have been conducted rigorously, with appropriate controls, replication, and sample sizes. The conclusions must be drawn appropriately based on the data presented.

Reviewer #1: Partly

Reviewer #2: Partly

Reviewer #3: Partly

**********

2. Has the statistical analysis been performed appropriately and rigorously?

Reviewer #1: N/A

Reviewer #2: Yes

Reviewer #3: No

**********

3. Have the authors made all data underlying the findings in their manuscript fully available?

The PLOS Data policy requires authors to make all data underlying the findings described in their manuscript fully available without restriction, with rare exception (please refer to the Data Availability Statement in the manuscript PDF file). The data should be provided as part of the manuscript or its supporting information, or deposited to a public repository. For example, in addition to summary statistics, the data points behind means, medians and variance measures should be available. If there are restrictions on publicly sharing data—e.g. participant privacy or use of data from a third party—those must be specified.

Reviewer #1: Yes

Reviewer #2: Yes

Reviewer #3: No

**********

4. Is the manuscript presented in an intelligible fashion and written in standard English?

PLOS ONE does not copyedit accepted manuscripts, so the language in submitted articles must be clear, correct, and unambiguous. Any typographical or grammatical errors should be corrected at revision, so please note any specific errors here.

Reviewer #1: Yes

Reviewer #2: Yes

Reviewer #3: Yes

**********

5. Review Comments to the Author

Please use the space provided to explain your answers to the questions above. You may also include additional comments for the author, including concerns about dual publication, research ethics, or publication ethics. (Please upload your review as an attachment if it exceeds 20,000 characters)

Reviewer #1: I congratulate the authors on their work. The study investigates how geographic proximity and linguistic relationships influence the composition and use of wild plants among 22 Indigenous groups in Mexico. The authors hypothesize that ethnofloras become more distinct with increasing geographic distance, and that culturally or linguistically related groups share more similar plant uses due to shared ancestral knowledge and interaction. Although the topic is highly interesting and the study presents extremely rich data and is well written, it lacks a stronger theoretical framework to demonstrate the expected relationship between geographic proximity, linguistic phylogeny, and ethnoflora.

My recommendation is that the authors review and strengthen the theoretical framework in the introduction and add a more robust discussion of their results. I reiterate that the study is highly relevant and should be published, but I believe that the data are currently underutilized in the way the manuscript is presented.

Below, I provide my specific comments on each topic.

Introduction

The authors mention only briefly, at the end, that geographic proximity may serve in the study as a proxy for an environmental factor influencing plant selection, while linguistic proximity may serve as a proxy for a cultural factor. This was the way I was able to interpret the issue, although it is not clearly stated in the text. I strongly suggest that the authors expand the introduction to outline the mechanisms through which geographic proximity could actually reflect environmental similarity or, in some cases, facilitate human mobility and thus the sharing of information (we might be observing either a convergence or divergence evolutionary process, depending on the case).

The same applies to the linguistic perspective. What evolutionary aspects underlie this cultural proxy? Is it a shared past and the broader phenomenon whereby linguistic divergence occurred alongside the differentiation of other cultural traits, such as local ecological knowledge? Or is it rather the ease of communication in more recent times? In that case, what is the role of the currently adopted colonial language? In summary, I felt the lack of a more comprehensive introduction that would help the reader fully understand the phenomenon under study.

I strongly recommend that the authors consult literature along the following lines, which may help them to develop a deeper and more coherent argumentative framework:

Currie, Thomas E.; Mace, Ruth. The evolution of ethnolinguistic diversity. Advances in Complex Systems, [S. l.], v. 15, n. 01n02, p. 1150006, 2012. DOI: 10.1142/S0219525911003372.

Hua, Xia; Greenhill, Simon J.; Cardillo, Marcel; Schneemann, Hilde; Bromham, Lindell. The ecological drivers of variation in global language diversity. Nature Communications, [S. l.], v. 10, n. 1, p. 2047, 2019. DOI: 10.1038/s41467-019-09842-2.

Moore, Joslin L.; Manne, Lisa; Brooks, Thomas; Burgess, Neil D.; Davies, Robert; Rahbek, Carsten; Williams, Paul; Balmford, Andrew. The distribution of cultural and biological diversity in Africa. Proceedings of the Royal Society of London. Series B: Biological Sciences, [S. l.], v. 269, n. 1501, p. 1645–1653, 2002. DOI: 10.1098/rspb.2002.2075.

Methods:

What is the rationale for choosing a cutoff of 40 records for inclusion in the database? Is there a statistical justification for this choice?

Another question: Did the authors consider reanalyzing the data by grouping together only those ethnic groups with a similar number of recorded species? Although the authors argue that the agreement between Simpson and Jaccard indices would demonstrate the absence of sampling bias, what methodological biases might still exist when comparing the dissimilarity of a group with 29 species to another with 660? Even if the 29 species are included within the set of 660, would they truly be considered completely similar? I recommend excluding outliers in terms of the number of species and re-running the analyses to verify whether dissimilarity decreases and how this might affect the results and discussion.

In addition, the authors should provide a clearer explanation of how the analysis of species composition and plant use categories was performed. Were these separated into information units such as plant–function pairs? The authors could include examples to help readers better understand how this categorization was carried out.

Results:

I found Table S1 difficult to interpret. Could the authors clarify what the names indicated at each level (e.g., “South,” “Pimic,” “Cahitan”) represent? Do they correspond to sub-branches or internal divisions within each language family?

Discussion:

There are divergent ideas in the discussion that make it difficult to fully understand the phenomenon. On the one hand, the authors suggest a possible environmental selection effect (as I proposed earlier as a theoretical pathway for the introduction), arguing that geographically close localities tend to have more similar ethnofloras. However, the text later reaffirms that all ethnofloras are very different from each other, even those that are geographically close. It is also mentioned that nearby localities can be ecologically very distinct, which contradicts the initial explanation. Without considering these specificities, it becomes unclear what the real pattern revealed by the analyses is. Again, I suggest reconsidering the analyses by removing the outliers to verify whether this could help clarify the observed trends.

I also suggest that the discussion be further developed regarding the possibility that plant selection is driven more by the environment than by language (with language serving as a cultural proxy). Although this is not entirely clear, it seems to be the pattern supported by the study.

I also believe that the study lacks a more detailed discussion of the fact that differences in dissimilarity with respect to geographic and linguistic separation are more pronounced when analyzed at the level of the complete information unit (plant–use) compared to analyses based solely on species diversity.

My final suggestion is that the authors consider resubmitting the work in the future, incorporating a stronger theoretical framework and a more thorough discussion to fully leverage the richness of the data and results.

Reviewer #2: Lines 63 to 66: When discussing biocultural knowledge and its relationship to alpha and beta diversity, there is no reference to support this. There are publications related to the topic.

Lines 118-125: It's important to mention the methodology used to obtain the information found in BADEPLAM. A reference alone isn't enough.

Lines 125-126: The methodology used to obtain the articles that were analyzed in the study is not mentioned. If the list of included studies was the result of a systematic review, it is important to include all stages of the process.

Reviewer #3: The manuscript is interesting and well written. However, several theoretical and methodological aspects are missing in order for it to be clear and to become an appropriate contribution for PLOS ONE. The authors are requested to address the following points:

1.The study analyzes changes in ethnofloras among human groups within Mexico. However, it does not compare its results with any other research conducted in other countries. It is necessary to present evidence of patterns observed regarding the turnover in the use of plant species at comparable scales, such as countries or regions. Accordingly, a substantial expansion of both the Introduction and Discussion sections is expected.

2. I consider the supplementary material insufficient. An essential element in any research article is the provision of sufficient methodological information to allow reproducibility. The “Supplementary list of references” (Supporting information S1.PDF) shows that a significant portion of the original sources are unpublished works corresponding to theses at different academic levels. Do the authors have the legal rights to use these sources, given that it is understood that they reproduce the extensive species lists compiled by those students? It is necessary that this ethical aspect be explicitly addressed by the authors and that it complies with PLOS ONE’s policies.

3. The authors do not provide sufficient data in their supplementary material, or at least they do not do in a format that can be easily reviewed anonymously. The authors provide an annex (https://doi.org/10.5281/zenodo.17238925

), although it is not possible to access it because doing so would compromise the anonymity of the reviewer. This may also be the case for future readers in the event the manuscript is published. It is therefore essential that the information be made openly available within the journal’s own platform. Furthermore, sufficient information must be provided so that the analyses can be replicated. In its current form, this point is unclear.

4. It is essential to explicitly state the confidence intervals obtained for the ethnoflora analyses for each Indigenous group reported. It is not clear whether further sampling is needed or whether the data compiled from different sources provide adequate sampling effort. The sampling effort must be specified for each Indigenous group included in the analysis. Curves indicating sampling effort and confidence intervals for the results for each Indigenous group are required. This must also be discussed.

This is an interesting study; however, it is considered that several aspects, such as those mentioned above, must be further developed in greater depth.

**********

6. PLOS authors have the option to publish the peer review history of their article (what does this mean?). If published, this will include your full peer review and any attached files.

If you choose “no”, your identity will remain anonymous but your review may still be made public.

**Do you want your identity to be public for this peer review?**   For information about this choice, including consent withdrawal, please see our Privacy Policy.

Reviewer #1: No

Reviewer #2: No

Reviewer #3: No

**********

[NOTE: If reviewer comments were submitted as an attachment file, they will be attached to this email and accessible via the submission site. Please log into your account, locate the manuscript record, and check for the action link "View Attachments". If this link does not appear, there are no attachment files.]

To ensure your figures meet our technical requirements, please review our figure guidelines: https://journals.plos.org/plosone/s/figures

You may also use PLOS’s free figure tool, NAAS, to help you prepare publication quality figures: https://journals.plos.org/plosone/s/figures#loc-tools-for-figure-preparation.

NAAS will assess whether your figures meet our technical requirements by comparing each figure against our figure specifications.

Attachment Submitted filename: Reviewe.docx

# Author comment: Author response to Decision Letter 1

**DOI:** 10.1371/journal.pone.0347334.r002

6 Feb 2026

RESPONSE TO REVIEWERS AND EDITOR

Editor comments:

*Some relevant studies addressing closely related ideas are not cited in the introduction. It would be helpful to briefly discuss their main findings and clarify how your study advances beyond them. For example: https://doi.org/10.1098/rspb. 2013.2768; https://doi.org/10.1016/j.jep.2014.12.007

RESPONSE: We appreciate this suggestion; these articles were very valuable in improving our interpretation of the data. The articles are now cited in the introduction and in the discussion. Our works are relatively similar, so it helps us confirm the patterns we observed.

*In the paragraph presenting your hypotheses, the distinction between plant composition and similarity in use remains somewhat unclear. Currently, only the hypothesis concerning geographic proximity is explicitly formulated, whereas the expected effect of linguistic relatedness on species composition is not clearly stated. Please revise this section to make both aspects explicit and conceptually distinct.

RESPONSE: We made some phrasing changes to better formulate the hypotheses of geographical and linguistic proximity and the relationship with the two approaches of this study; species composition and species uses among ethnofloras. The changes made to the introduction, reinforcing the theoretical framework, also contribute to a better explanation of the hypotheses.

Reviewer A

*My recommendation is that the authors review and strengthen the theoretical framework in the introduction and add a more robust discussion of their results. I reiterate that the study is highly relevant and should be published, but I believe that the data are currently underutilized in the way the manuscript is presented.

RESPONSE: Thank you for your congratulations, and we hope we have improved the article with your valuable feedback.

Introduction

*The authors mention only briefly, at the end, that geographic proximity may serve in the study as a proxy for an environmental factor influencing plant selection, while linguistic proximity may serve as a proxy for a cultural factor. This was the way I was able to interpret the issue, although it is not clearly stated in the text. I strongly suggest that the authors expand the introduction to outline the mechanisms through which geographic proximity could actually reflect environmental similarity or, in some cases, facilitate human mobility and thus the sharing of information (we might be observing either a convergence or divergence evolutionary process, depending on the case).

The same applies to the linguistic perspective. What evolutionary aspects underlie this cultural proxy? Is it a shared past and the broader phenomenon whereby linguistic divergence occurred alongside the differentiation of other cultural traits, such as local ecological knowledge? Or is it rather the ease of communication in more recent times? In that case, what is the role of the currently adopted colonial language? In summary, I felt the lack of a more comprehensive introduction that would help the reader fully understand the phenomenon under study.

I strongly recommend that the authors consult literature along the following lines, which may help them to develop a deeper and more coherent argumentative framework:

RESPONSE: We appreciate your suggestions. The literature you and the editor shared helped us strengthen our theoretical framework on geographic and linguistic processes. Taking your observation into account, the introduction now includes information that better supports the theoretical framework explaining how geographic proximity and linguistic phylogeny, proxies for the cultural and floristic affinities are explained variations in the uses and species composition of ethnofloras.

Methods:

*What is the rationale for choosing a cutoff of 40 records for inclusion in the database? Is there a statistical justification for this choice?

RESPONSE: When a system (in this case an ethnic group) has very few data, a few unusual species can lead to severe misclassification (in cluster analyses) or to poor estimates of dissimilarity. Thus, it is common practice to eliminate such data (Jongman, R.H.G., Ter Braak, C.J. and Van Tongeren, O.F. eds., 1995. Data analysis in community and landscape ecology. Cambridge university press). This information and reference has been added to the main text.

*Another question: Did the authors consider reanalyzing the data by grouping together only those ethnic groups with a similar number of recorded species? Although the authors argue that the agreement between Simpson and Jaccard indices would demonstrate the absence of sampling bias, what methodological biases might still exist when comparing the dissimilarity of a group with 29 species to another with 660? Even if the 29 species are included within the set of 660, would they truly be considered completely similar? I recommend excluding outliers in terms of the number of species and re-running the analyses to verify whether dissimilarity decreases and how this might affect the results and discussion.

RESPONSE: This is a very nice idea and addresses also the concerns of reviewer C about incomplete sampling. We repeated our analyses after removing all ethnic groups with only one and with more than 5 studies and found the same results as with the Simpson index.

However, we can do better that this and take advantage of all our data without dropping information. Ethnicities can be grouped based on the number of species recorded for each one or the number of publications that we consulted. The Mantel correlations can then be calculated for each data subset. Because the subsets would thus have far less variability in terms of sampling effort, any bias that it may introduce in the correlations would be strongly reduced. Because the individual Mantel statistics are testing the same null hypothesis using different datasets, they can then be merged into a single test via the Fisher’s method (Gotelli, N.J. and Ellison, A.M. 2013. A Primer of Ecological Statistics. Sinauer, Sunderland; Shipley, B., 2016. Cause and correlation in biology: A user's guide to path analysis, structural equations and causal inference with R. Cambridge university press.). Again, such grouping by number of species or of publications produced the same results as the Mantel analysis based on the Simpson index. The fact that three different statistical procedures give the same outcome suggests that our results are in fact robust to incomplete-sampling bias. The methodological description and the results table were added to the main text.

* In addition, the authors should provide a clearer explanation of how the analysis of species composition and plant use categories was performed. Were these separated into information units such as plant–function pairs? The authors could include examples to help readers better understand how this categorization was carried out.

RESPONSE: We have provided a better description of the method and indicated the path in the repository (Zenodo) where the matrices can be consulted. Due to a misunderstanding regarding the PLOS ONE instructions, we did not provide open access to Zenodo. We will now provide a Google Drive link so that reviewers can anonymously examine the matrices, and if the article is accepted, access to Zenodo will be made public. We believe that once you have access to the matrices (SppEtnia_Jaccard.csv and usos_Jaccard.csv), it will be clear how the data were organized for the species composition and land use analyses.

Results:

*I found Table S1 difficult to interpret. Could the authors clarify what the names indicated at each level (e.g., “South,” “Pimic,” “Cahitan”) represent? Do they correspond to sub-branches or internal divisions within each language family?

RESPONSE: The language spoken by each ethnic group is classified into subgroups (levels 2 to 4) that belong to a main language family (level 1). This measures the degree of intelligibility of the languages. The names of each variant come from how these groups named their languages or how they are named according to the classification established by linguists. To be clearer, in the methods section we have added an explanation of this hierarchical division and also made improvements to the table and its caption. This table now includes a color-coded guide to represent the hierarchical relationship of the languages. We also considered placing the table within the main text so that the description of the linguistic distance estimation would be closer to the table used for the calculation.

Discussion:

*There are divergent ideas in the discussion that make it difficult to fully understand the phenomenon. On the one hand, the authors suggest a possible environmental selection effect (as I proposed earlier as a theoretical pathway for the introduction), arguing that geographically close localities tend to have more similar ethnofloras. However, the text later reaffirms that all ethnofloras are very different from each other, even those that are geographically close. It is also mentioned that nearby localities can be ecologically very distinct, which contradicts the initial explanation. Without considering these specificities, it becomes unclear what the real pattern revealed by the analyses is. Again, I suggest reconsidering the analyses by removing the outliers to verify whether this could help clarify the observed trends.

I also suggest that the discussion be further developed regarding the possibility that plant selection is driven more by the environment than by language (with language serving as a cultural proxy). Although this is not entirely clear, it seems to be the pattern supported by the study.

RESPONSE: We understand the confusion and have strived to present the arguments in the discussion more clearly. We believe that the improvements since the introduction help to better understand the effect of beta diversity, which shows the high percentage of turnover among ethnofloras because of Mexico's floristic characteristics. Therefore, the Jaccard dissimilarity values are high (0.8 to 1), indicating that the ethnofloras are not very similar. Nevertheless, the pattern is significant (as verified by Mantel's correlation using the four suggested methods), and we can say that the species composition of the ethnofloras is correlated with geographic and linguistic proximity, and the uses of species are correlated with geographic proximity and marginally with language. Therefore, in the discussion, we consider it important to explain the effects of the environment and language because, although to different degrees, both explain variations in species composition and uses.

To improve clarity, we made changes to the organization of the writing that we hope will help to better discuss the results.

*I also believe that the study lacks a more detailed discussion of the fact that differences in dissimilarity with respect to geographic and linguistic separation are more pronounced when analyzed at the level of the complete information unit (plant–use) compared to analyses based solely on species diversity.

RESPONSE: The reason for the greater similarity in the uses of species is that language is not a barrier and allows for greater consensus among ethnic groups regarding the uses they assign to species. This argument is found in the discussion between lines 359 and 363.

Reviewer B

Lines 63 to 66: When discussing biocultural knowledge and its relationship to alpha and beta diversity, there is no reference to support this. There are publications related to the topic.

RESPONSE: We didn't include a reference because we couldn't find one that presented a similar idea. In this case, we are proposing this analogy between alpha-beta ecological diversity and the way knowledge about biocultural diversity is distributed. However, in that section we have added a reference that explains the ecological terms alpha and beta.

Lines 118-125: It's important to mention the methodology used to obtain the information found in BADEPLAM. A reference alone isn't enough.

RESPONSE: Thank you for your observation. To address your comment, we have included information in the methodology that more clearly describes the process by which BADEPLAM's information is obtained. We have clarified that it has been a historical and ongoing process of compiling and curating data on useful plants, obtained from publicly available bibliographic sources. We also clarify that this database is currently under our management, and therefore we are responsible for its curation. The methodology section describes how the scientific names of the species are curated to keep the names up to date.

Lines 125-126: The methodology used to obtain the articles that were analyzed in the study is not mentioned. If the list of included studies was the result of a systematic review, it is important to include all stages of the process.

RESPONSE: We appreciate your feedback, as it helps us better communicate our methodology. The data used for this article was already captured and curated in BADEPLAM and comes from the articles listed in the supplementary section. The selection of bibliographic sources in BADEPLAM is the result of a historical and continuous search process that has been ongoing for 40 years. As mentioned in the previous response, this explanation is found in the method section, where we aim to explain how the people in charge of the database have selected their sources over this period.

Reviewer C

The manuscript is interesting and well written. However, several theoretical and methodological aspects are missing in order for it to be clear and to become an appropriate contribution for PLOS ONE. The authors are requested to address the following points:

1.The study analyzes changes in ethnofloras among human groups within Mexico. However, it does not compare its results with any other research conducted in other countries. It is necessary to present evidence of patterns observed regarding the turnover in the use of plant species at comparable scales, such as countries or regions. Accordingly, a substantial expansion of both the Introduction and Discussion sections is expected.

RESPONSE: We appreciate your observation and have placed greater emphasis on finding other research that has studied the intercultural variation of ethnofloras at the scale we did, incorporating cultural and geo-floristic processes. Research by Saslis-Lagoudakis, which we had not previously reviewed, particularly the work published in 2014, shares many attributes with our research. This article, along with others shared by the editor and reviewers, are cited in the introduction and discussion to enhance the theoretical framework and allow for comparisons with other regions such as Nepal or the Basque Country in Spain.

The analysis of floristic turnover in ethnofloras is a proposal we put forward in this article, but we have not found a similar analysis in another research. Saslis-Lagoudakis et al. (2014) estimate differences in the ethnic groups´ medicinal floras and floristic environments in Nepal using phylogenetic information of the flora, but not beta diversity. Therefore, we have no citations from other regions worldwide.

2. I consider the supplementary material insufficient. An essential element in any research article is the provision of sufficient methodological information to allow reproducibility. The “Supplementary list of references” (Supporting information S1.PDF) shows that a significant portion of the original sources are unpublished works corresponding to theses at different academic levels. Do the authors have the legal rights to use these sources, given that it is understood that they reproduce the extensive species lists compiled by those students? It is necessary that this ethical aspect be explicitly addressed by the authors and that it complies with PLOS ONE’s policies.

RESPONSE: We appreciate your observation, therefore the description of the information contained in the database (BADEPLAM) (see Methodology) explicitly states that the data obtained from theses, books and articles are public data. This is stated in the article:

“For four decades, information of Mexican useful plants species has been continuously compiled and curated in

Attachment Submitted filename: RESPON~1.DOC

# Editor report: Decision Letter 1

**DOI:** 10.1371/journal.pone.0347334.r003

**Authors:** Washington Ferreira Júnior, Washington Ferreira Júnior

23 Feb 2026

PONE-D-25-53514R1

Biodiversity drives the choice; linguistic diversity fine-tunes the direction: Ethnofloral megadiversity in the Mexican ethnobotany.

PLOS One

Dear Dr. Martínez-Ballesté,

Thank you for submitting your manuscript to PLOS ONE. After careful consideration, we feel that it has merit but does not fully meet PLOS ONE’s publication criteria as it currently stands. Therefore, we invite you to submit a revised version of the manuscript that addresses the points raised during the review process.

Please submit your revised manuscript by Apr 09 2026 11:59PM. If you will need more time than this to complete your revisions, please reply to this message or contact the journal office at plosone@plos.org. When you're ready to submit your revision, log on to https://www.editorialmanager.com/pone/ and select the 'Submissions Needing Revision' folder to locate your manuscript file.

Please include the following items when submitting your revised manuscript:

- A letter that responds to each point raised by the academic editor and reviewer(s). You should upload this letter as a separate file labeled 'Response to Reviewers'.
- A marked-up copy of your manuscript that highlights changes made to the original version. You should upload this as a separate file labeled 'Revised Manuscript with Track Changes'.
- An unmarked version of your revised paper without tracked changes. You should upload this as a separate file labeled 'Manuscript'.

-->If you would like to make changes to your financial disclosure, please include your updated statement in your cover letter. Guidelines for resubmitting your figure files are available below the reviewer comments at the end of this letter.

If applicable, we recommend that you deposit your laboratory protocols in protocols.io to enhance the reproducibility of your results. Protocols.io assigns your protocol its own identifier (DOI) so that it can be cited independently in the future. For instructions see: https://journals.plos.org/plosone/s/submission-guidelines#loc-laboratory-protocols. Additionally, PLOS ONE offers an option for publishing peer-reviewed Lab Protocol articles, which describe protocols hosted on protocols.io. Read more information on sharing protocols at https://plos.org/protocols?utm_medium=editorial-email&utm_source=authorletters&utm_campaign=protocols.

We look forward to receiving your revised manuscript.

Kind regards,

Washington Soares Ferreira Júnior

Academic Editor

PLOS One

Journal Requirements:

If the reviewer comments include a recommendation to cite specific previously published works, please review and evaluate these publications to determine whether they are relevant and should be cited. There is no requirement to cite these works unless the editor has indicated otherwise.

Please review your reference list to ensure that it is complete and correct. If you have cited papers that have been retracted, please include the rationale for doing so in the manuscript text, or remove these references and replace them with relevant current references. Any changes to the reference list should be mentioned in the rebuttal letter that accompanies your revised manuscript. If you need to cite a retracted article, indicate the article’s retracted status in the References list and also include a citation and full reference for the retraction notice.

Additional Editor Comments:

I would like to begin by acknowledging the considerable effort the authors have made in revising the manuscript. The current version represents a substantial improvement over the previous one. The authors have responded carefully and thoughtfully to the reviewers’ comments, and most of the suggested revisions have been satisfactorily addressed. The manuscript is now clearer, methodologically more transparent, and conceptually more structured. In particular, I appreciate the additional analyses conducted to control for potential sampling biases and the expanded explanations regarding methodological decisions. These additions have strengthened the robustness of the study and improved the clarity of the analytical framework.

That said, the process of detailing important methodological and interpretative aspects has also raised a few additional points that merit further clarification. These are not major flaws, but rather issues of precision and interpretation that, if addressed, would further enhance the rigor and coherence of the manuscript.

First, some of the interpretative language in the discussion occasionally appears stronger than what the statistical analyses strictly allow. Given that the Mantel and partial Mantel tests assess correlations rather than causal relationships, I recommend carefully revising statements that imply determination or causality. Framing the results in terms of statistically supported associations would ensure greater conceptual precision and avoid overinterpretation.

Second, although the authors have made a commendable effort to address sampling effort concerns, particularly regarding alpha diversity, there remain important limitations inherent to the use of heterogeneous bibliographic sources collected over many decades. In this regard, I encourage the authors to explicitly consider the temporal dimension as a potential source of bias. Some areas may be represented primarily by older studies, while others are documented mostly by recent research. Likewise, for the same area, combining studies conducted in very different time periods may introduce a temporal bias, since ethnofloristic knowledge and species use can change substantially over time due to ecological transformations, socioeconomic shifts, or cultural dynamics. A more explicit acknowledgment of this temporal heterogeneity, and its possible implications for the observed patterns, would strengthen the discussion of limitations.

Third, I would like to draw attention to the formulation of the hypothesis. In its current wording, the hypothesis appears to conflate distinct mechanisms within a single explanatory framework. However, these components operate through partially different processes and would benefit from clearer analytical separation. A more precise formulation would distinguish between (1) similarity in the composition of wild plant species used by different ethnic groups and (2) similarity in the uses attributed to those species. These dimensions may be positively associated with (i) geographic proximity, reflecting greater similarity in the available flora in spatially close regions, and (ii) linguistic proximity, reflecting shared cultural ancestry and/or greater ease of knowledge transmission among linguistically related groups. Importantly, as acknowledged in earlier sections of the Introduction, geographic and linguistic proximity are themselves spatially correlated. For this reason, their effects should be treated as partially independent and statistically disentangled. Clarifying the hypothesis along these lines would strengthen its conceptual coherence by explicitly separating ecological constraints from cultural transmission processes, distinguishing species composition from patterns of use, and aligning the theoretical expectations more closely with the analytical framework employed in the study.

Overall, I believe this study makes an important contribution to understanding ethnofloristic diversity and its ecological and cultural correlates. The manuscript is now much improved, and with minor revisions focused on interpretative precision and conceptual clarity, it will be further strengthened. I appreciate the authors’ dedication to refining the work and encourage them to consider these final adjustments in the next version.

[NOTE: If reviewer comments were submitted as an attachment file, they will be attached to this email and accessible via the submission site. Please log into your account, locate the manuscript record, and check for the action link "View Attachments". If this link does not appear, there are no attachment files.]

To ensure your figures meet our technical requirements, please review our figure guidelines: https://journals.plos.org/plosone/s/figures

You may also use PLOS’s free figure tool, NAAS, to help you prepare publication quality figures: https://journals.plos.org/plosone/s/figures#loc-tools-for-figure-preparation.

NAAS will assess whether your figures meet our technical requirements by comparing each figure against our figure specifications.

# Author comment: Author response to Decision Letter 2

**DOI:** 10.1371/journal.pone.0347334.r004

25 Mar 2026

RESPONSE TO EDITOR COMMENTS

First, some of the interpretative language in the discussion occasionally appears stronger than what the statistical analyses strictly allow. Given that the Mantel and partial Mantel tests assess correlations rather than causal relationships, I recommend carefully revising statements that imply determination or causality. Framing the results in terms of statistically supported associations would ensure greater conceptual precision and avoid overinterpretation.

Response: Thank you for your observation. We have carefully reviewed the language of our interpretations in the discussion and softened it into two sentences (lines 339-340; 348-349) where we now explain the results as associations rather than causal relationships.

Second, although the authors have made a commendable effort to address sampling effort concerns, particularly regarding alpha diversity, there remain important limitations inherent to the use of heterogeneous bibliographic sources collected over many decades. In this regard, I encourage the authors to explicitly consider the temporal dimension as a potential source of bias. Some areas may be represented primarily by older studies, while others are documented mostly by recent research. Likewise, for the same area, combining studies conducted in very different time periods may introduce a temporal bias, since ethnofloristic knowledge and species use can change substantially over time due to ecological transformations, socioeconomic shifts, or cultural dynamics. A more explicit acknowledgment of this temporal heterogeneity, and its possible implications for the observed patterns, would strengthen the discussion of limitations.

Response: We understand your argument and agree that the differences in the years covered by the literature used can bias the interpretation. Since we do not have studies that are consistently repeated across different years for all ethnicities, it was acknowledged at the end of the discussion (lines 389-396) that we lack this type of data to analyze this temporal variation in this research. However, we also argued in the discussion that we could assume there are not many changes, as we found that 87% of the citations we used correspond to a modern four-decade period (1980-2017), and only 11 references cover the period from 1935 to 1979. This distribution of citations across the years was also explained in the methodology section (lines 166-167).

Third, I would like to draw attention to the formulation of the hypothesis. In its current wording, the hypothesis appears to conflate distinct mechanisms within a single explanatory framework. However, these components operate through partially different processes and would benefit from clearer analytical separation. A more precise formulation would distinguish between (1) similarity in the composition of wild plant species used by different ethnic groups and (2) similarity in the uses attributed to those species. These dimensions may be positively associated with (i) geographic proximity, reflecting greater similarity in the available flora in spatially close regions, and (ii) linguistic proximity, reflecting shared cultural ancestry and/or greater ease of knowledge transmission among linguistically related groups. Importantly, as acknowledged in earlier sections of the Introduction, geographic and linguistic proximity are themselves spatially correlated. For this reason, their effects should be treated as partially independent and statistically disentangled. Clarifying the hypothesis along these lines would strengthen its conceptual coherence by explicitly separating ecological constraints from cultural transmission processes, distinguishing species composition from patterns of use, and aligning the theoretical expectations more closely with the analytical framework employed in the study.

Response: We appreciate your suggestions for better explaining the objectives and hypotheses of this research. We have taken your suggestions into account, and the changes can be found at the end of the introduction (lines 137-145).

Attachment Submitted filename: Response to Reviewers2.docx

# Editor report: Decision Letter 2

**DOI:** 10.1371/journal.pone.0347334.r005

**Authors:** Washington Ferreira Júnior, Washington Ferreira Júnior, Washington Ferreira Júnior

1 Apr 2026

Biodiversity drives the choice; linguistic diversity fine-tunes the direction: Ethnofloral megadiversity in the Mexican ethnobotany.

PONE-D-25-53514R2

Dear Dr. Andrea Martínez-Ballesté,

We’re pleased to inform you that your manuscript has been judged scientifically suitable for publication and will be formally accepted for publication once it meets all outstanding technical requirements.

Within one week, you’ll receive an e-mail detailing the required amendments. When these have been addressed, you’ll receive a formal acceptance letter and your manuscript will be scheduled for publication.

An invoice will be generated when your article is formally accepted. Please note, if your institution has a publishing partnership with PLOS and your article meets the relevant criteria, all or part of your publication costs will be covered. Please make sure your user information is up-to-date by logging into Editorial Manager at Editorial Manager® and clicking the ‘Update My Information' link at the top of the page. For questions related to billing, please contact billing support.

If your institution or institutions have a press office, please notify them about your upcoming paper to help maximize its impact. If they’ll be preparing press materials, please inform our press team as soon as possible -- no later than 48 hours after receiving the formal acceptance. Your manuscript will remain under strict press embargo until 2 pm Eastern Time on the date of publication. For more information, please contact onepress@plos.org.

Kind regards,

Washington Soares Ferreira Júnior

Academic Editor

PLOS One

# Editor report: Acceptance letter

**DOI:** 10.1371/journal.pone.0347334.r006

**Authors:** Washington Ferreira Júnior, Washington Ferreira Júnior, Washington Ferreira Júnior

PONE-D-25-53514R2

PLOS One

Dear Dr. Martínez-Ballesté,

I'm pleased to inform you that your manuscript has been deemed suitable for publication in PLOS One. Congratulations! Your manuscript is now being handed over to our production team.

At this stage, our production department will prepare your paper for publication. This includes ensuring the following:

* All references, tables, and figures are properly cited

* All relevant supporting information is included in the manuscript submission,

* There are no issues that prevent the paper from being properly typeset

You will receive further instructions from the production team, including instructions on how to review your proof when it is ready. Please keep in mind that we are working through a large volume of accepted articles, so please give us a few days to review your paper and let you know the next and final steps.

Lastly, if your institution or institutions have a press office, please let them know about your upcoming paper now to help maximize its impact. If they'll be preparing press materials, please inform our press team within the next 48 hours. Your manuscript will remain under strict press embargo until 2 pm Eastern Time on the date of publication. For more information, please contact onepress@plos.org.

You will receive an invoice from PLOS for your publication fee after your manuscript has reached the completed accept phase. If you receive an email requesting payment before acceptance or for any other service, this may be a phishing scheme. Learn how to identify phishing emails and protect your accounts at https://explore.plos.org/phishing.

If we can help with anything else, please email us at customercare@plos.org.

Thank you for submitting your work to PLOS ONE and supporting open access.

Kind regards,

PLOS ONE Editorial Office Staff

on behalf of

Dr. Washington Soares Ferreira Júnior

Academic Editor

PLOS One
